# Compromised repolarization reserve in a murine model of catecholaminergic polymorphic ventricular tachycardia caused by RyR2-R420Q mutation

**DOI:** 10.1016/j.yjmcc.2025.07.014

**Published:** 2025-07-24

**Authors:** Spyros Zissimopoulos, Pavel Kirilenko, Aitana Braza-Boïls, Esther Zorio, Yueyi Wang, Ana Maria Gomez, Mark B. Cannell, Branko Latinkic, Ewan D. Fowler

**Affiliations:** a Institute of Life Science, Swansea University Medical School, Swansea University, Swansea SA2 8PP, UK; b School of Biosciences, College of Biomedical and Life Sciences, Cardiff University, Cardiff CF10 3AX, UK; c CAFAMUSME Research Group, Instituto de Investigación Sanitaria La Fe de Valencia, Spain; d CIBERCV, Center for Biomedical Network Research on Cardiovascular Diseases, Madrid, Spain; e Inherited Cardiac Diseases Unit, Cardiology Department at the Hospital Universitario y Politécnico La Fe de Valencia, Spain; f Department of Medicine, Faculty of Medicine and Dentistry, Universitat de València, 46010, Valencia, Spain; g Inserm, UMR-S 1180, Signalling and Cardiovascular Pathophysiology, Faculté de Pharmacie, Université Paris-Saclay, 17 Avenue des Sciences, 91400 Orsay, France; h School of Physiology, Pharmacology & Neuroscience, Faculty of Biomedical Sciences, University of Bristol, University Walk, Bristol BS8 1TD, UK

**Keywords:** CPVT, Arrhythmias, Early afterdepolarizations, Calcium sparks, Repolarization reserve

## Abstract

**Background:**

Catecholaminergic polymorphic ventricular tachycardia (CPVT) is a malignant inherited heart disease characterised by stress-induced arrhythmias that are thought to be caused by delayed after-depolarizations resulting from abnormal Ca^2+^ cycling. Some patients exhibit unusually large ECG U-waves that could be associated with altered ventricular repolarization, but the possible link with dysfunctional RyR2 is unclear. We investigated whether increased Ca^2+^ leak during systole disrupts repolarization in a transgenic mouse model of CPVT.

**Methods:**

Electrocardiograms were recorded in patients with RyR2-R420Q CPVT mutation (R420Q). Experiments were performed on control and R420Q knock-in mouse hearts and ventricular myocytes.

**Results:**

R420Q patients had larger resting U-waves than family member controls. R420Q mouse hearts exhibited greater prolongation of monophasic APs following pauses in pacing and during beta-adrenergic stimulation. Ventricular ectopic beats during repolarization were more prevalent in R420Q mouse hearts following pacing-pauses and during premature electrical stimulation. Early afterdepolarizations (EADs) occurred in isolated R420Q myocytes during beta-adrenergic stimulation and coincided with increased Ca^2+^ leak during the Ca^2+^ transient decay, in the form of late Ca^2+^ sparks (LCS). AP voltage clamp electrophysiology experiments, analysis of LCS recovery, and computer simulations of hyperactive RyR2 supported a mechanism involving increased RyR2 sensitivity and/or reduced refractoriness that increased LCS frequency and inward sodium/calcium exchange current, resulting in AP prolongation and EADs.

**Conclusions:**

Ca^2+^-mediated AP lengthening and EADs may contribute to proarrhythmic behaviour in CPVT caused by gain-of-function R420Q mutation. Loss of repolarization reserve is not specifically targeted by CPVT therapies but could be an opportunity for therapeutic intervention.

## 1 Introduction

Catecholaminergic polymorphic ventricular tachycardia (CPVT) is a malignant inherited cardiac disease characterised by ventricular tachyarrhythmias that carries increased risk of sudden death brought on by exercise or emotional stress. [1,2] Mutations in the ryanodine receptor (RyR2) Ca^2+^ release channel have been identified in more than 50 % of CPVT patients and carry an autosomal dominant inheritance pattern. [3] CPVT is normally associated with hyperactive (“leaky”) RyR2, [4] causing increased Ca^2+^ spark rate and Ca^2+^ wave initiation in cardiac myocytes. Delayed afterdepolarizations (DADs) caused by diastolic Ca^2+^ waves are thought to be the underlying mechanism for arrhythmias and ventricular tachycardia in both preclinical models [5–7] and CPVT patients. [8] Current treatments for CPVT, such as beta-blockers and/or flecainide, are aimed at reducing DADs but do not provide complete protection against adverse cardiac events in all patients, [9] so a better understanding of the cellular mechanisms is needed.

Bidirectional ventricular tachycardia reportedly arose from late coupled extrasystolic beats in both patients and transgenic mice carrying the RyR2-R4496C^+/−^ CPVT mutation, [10] which would be consistent with a diastolic DAD-mediated mechanism. However, other ECG abnormalities have been reported in some CPVT patients, such as biphasic or notched T-wave morphology [11,12] and increased U-wave amplitude. [13,14] The cause of U-waves in humans is not fully understood and there may be overlap between mechanisms in different diseases. [15] U-waves are commonly associated with dysfunction of membrane ion channels and prolonged repolarization in conditions such as the long QT syndrome, [16] but these ionic currents are not thought to be altered in CPVT. Transmural repolarization gradients could alter U-wave morphology, and quantitative differences in repolarization gradients in mice versus larger mammals might explain why mice do not normally exhibit U-waves. [17] DADs were also shown to cause U-waves in canine ventricular wedge preparations. [18] Diastolic Ca^2+^ sparks and Ca^2+^ waves are normally suppressed during an apparent refractory period following a Ca^2+^ transient, [19,20] so it is unclear whether they can explain short-coupled abnormal T- and U-waves.

It was previously reported that patients with RyR2-R420Q^+/−^ (R420Q) mutation exhibited augmented U-waves and that isolated myocytes from R420Q knock-in mice had increased spontaneous dia-stolic Ca^2+^ release. [13] In this study, we sought to establish how dys-regulation of intracellular Ca^2+^ handling caused by this mutation impacts electrophysiology at the cellular and organ level, particularly whether it could contribute to abnormal repolarization. We investigated the inducibility of arrhythmias in intact perfused hearts and performed patch clamp electrophysiology and confocal Ca^2+^ imaging in isolated ventricular myocytes from R420Q mice. Mouse is currently the only mammalian model of human CPVT caused by gain-of-function RyR2, [5] but the mouse heart differs from human in some key areas, such as reliance on repolarizing K^+^ currents, action potential duration (APD) and morphology and consequently impact of Na^+^/Ca^2+^ exchange current (I_NCX_) on APD. Mouse ventricular myocytes share most of the Ca^2+^ handling mechanisms with humans and the R420Q mouse model replicates some of the electrophysiological features of the human disease, including a similar rate of ventricular tachycardia under basal conditions (compared to wildtype controls), but a greater incidence of arrhythmias in response to pharmacological or emotional stress. [21,22] Transgenic mice have also been used to study both DAD and early afterdepolarization (EAD) mechanisms, such as in Andersen-Tawil syndrome. [23] Our results indicate that RyR2 hypersensitivity increases Ca^2+^ leak during systole that contributes to AP lengthening and susceptibility to arrhythmias arising during ventricular repolarization in CPVT.

## 2 Methods

Detailed methods are available in the online Supplementary Materials. Experiments were conducted with local ethical approval in accordance with UK Home Office, French Ministry of Sciences (APAFIS#1297–2,015,072,114,403,692) and European Parliament Directive 2010/63/EU guidelines on the use of animals in research. Clinical evaluation of CPVT patients was conducted after signing informed consent in accordance with the Declaration of Helsinki and with the approval of the local Institutional Ethics Committee at the in Hospital Universitario y Politécnico La Fe de Valencia, Spain (#2018/0365). Detailed clinical characterisation of CPVT-causing RyR2 mutations used in this study were described in previous reports. [13,21,24]

## 3 Results

**Fig. 1:**
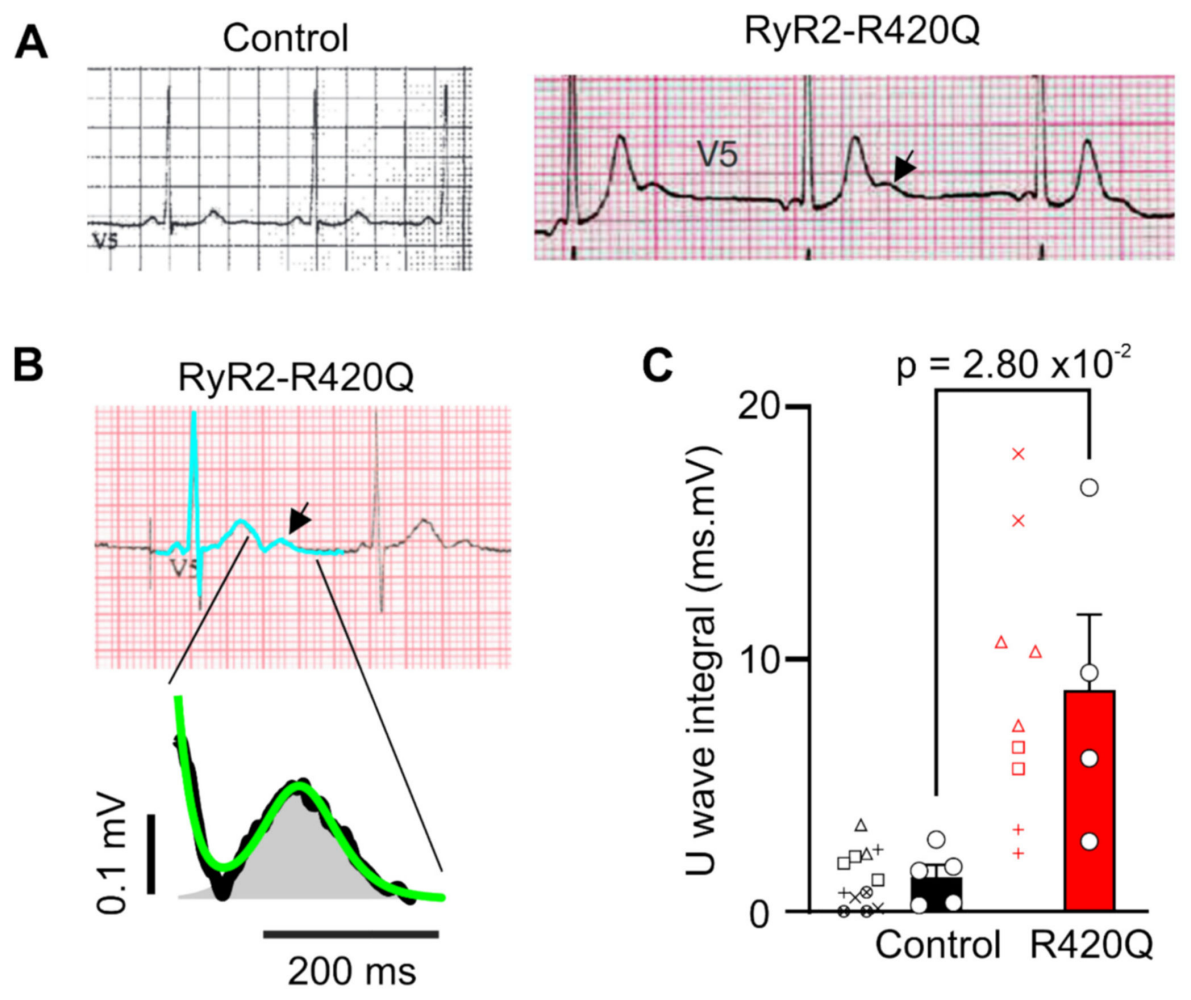
Increased ECG U-waves in RyR2-R420Q patients. A Resting ECG recordings from an RyR2-R420Q patient (right panel) and a genotype-negative family member control (left panel). Arrow indicates the large U-waves that occurred immediately following, and merging with, the preceding T-wave. B Resting ECG in a different R420Q patient. Method for analysing the T-wave and U-wave (shaded area) components of the TU complex by fitting the sum of a decaying exponential and Gaussian curve (green line), to digitized ECG recordings, as described in [25]. C The U-wave integral was greater in R420Q patients. Statistical analysis was conducted on the mean U-wave integral for each patient or control (open circles and bars) from the average of 1–3 cycles (individual cycle values from each patient or control are shown with different symbols beside the histogram bars). (C) unpaired *t*-test. N = 5 genotype negative family member controls and N = 4 R420Q patients. (For interpretation of the references to colour in this figure legend, the reader is referred to the web version of this article.)

Table 1 summarises resting ECG parameters in patients with the CPVT-causing R420Q mutation in the N-terminus of RyR2 (RyR2-R420Q) and genotype negative family members. QT and QTc intervals were not different between groups at rest. Fig. 1A shows an exemplar resting ECG recording from an R420Q patient with prominent U-waves that occurred immediately after, and merged with, the preceding T-wave, forming a TU complex. To analyse the U-wave component, digitized recordings of the TU complex were fitted with the sum of a decaying exponential and a Gaussian curve (Fig. 1B), following the method of Reilly et al. [25] The U-wave integral calculated using this method was greater in R420Q patients compared to controls (Fig. 1C). Large U-waves have also been reported with RyR2 mutations in other regions of the channel, such as the helical domain (RyR2-C2277R) (Fig. S1), [24] N-terminus domain (RyR2-R169Q), [26] bridging solenoid B (RyR2-P2328S), [11] and exon-3 deletion, [27] indicating that U-waves are not unique to the R420Q mutation examined here.

### 3.1 Prolonged action potential repolarization in R420Q hearts

**Fig. 2:**
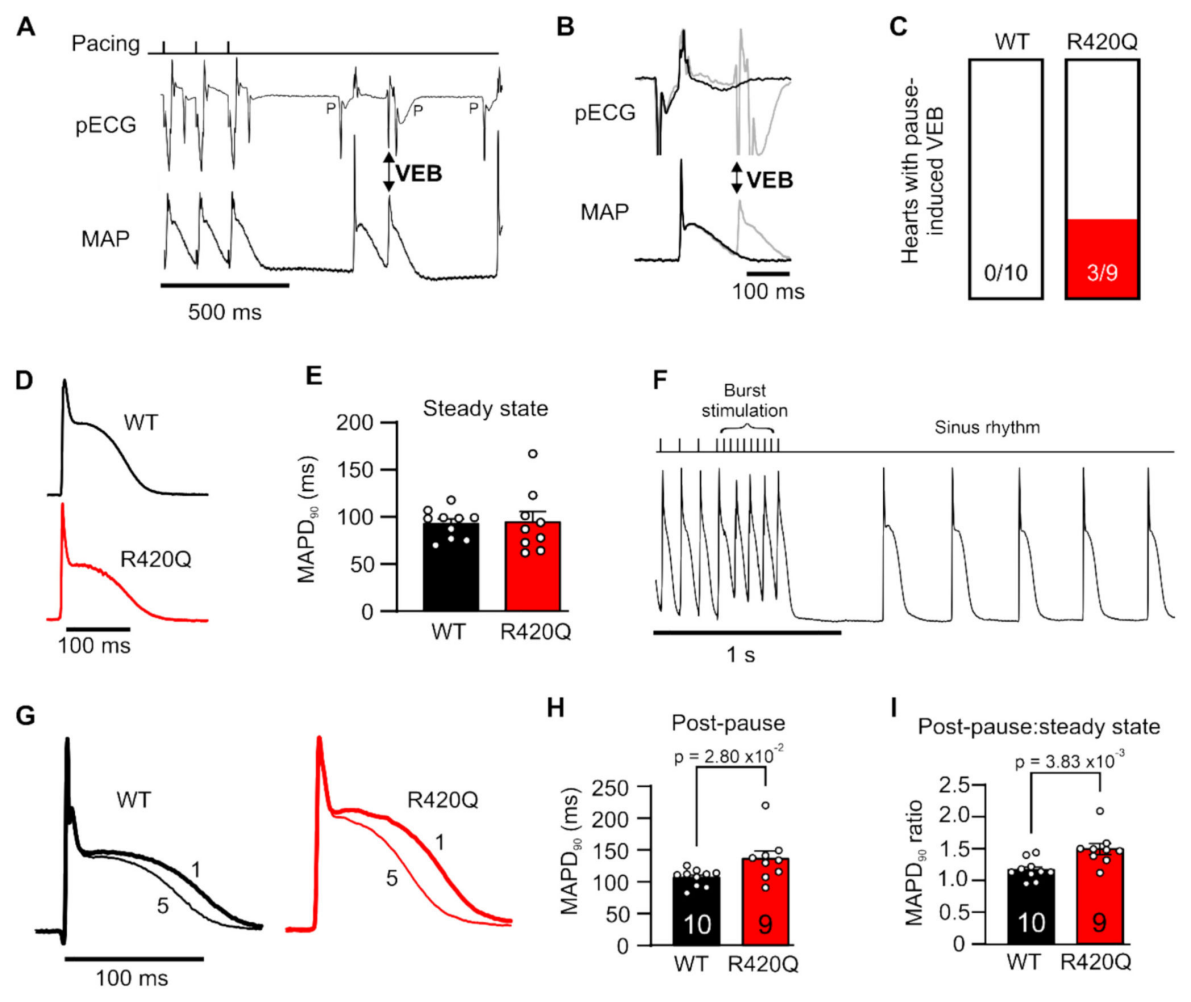
Pause-dependent ventricular ectopic beats (VEB) and AP lengthening in transgenic R420Q mouse hearts. A Monophasic action potential (MAP) and pseudo-ECG recording (pECG) measured simultaneously in an R420Q heart. When electrical pacing was turned off (top panel) there was a pause before sinus rhythm resumed, evident by the presence of P-waves (P). A VEB occurred during repolarization of the first sinus beat after the pause B Grey lines show the VEB in (A) overlaid with a different beat from the same heart that repolarizes normally on an expanded time scale (black lines). The VEB coincides with a premature ventricular complex on the ECG (double arrow), confirming this was not an artefact of MAP recording. C Pause-induced VEB occurred in 3/9 R420Q and 0/10 WT hearts. D Exemplar sinus rhythm MAP from a WT and R420Q heart. E Sinus rhythm MAPD_90_ was not different between WT and R420Q hearts. F Exemplar recording from an R420Q heart during and after burst pacing. G Exemplar MAP recordings from a WT and R420Q heart showing the first (thick line) and fifth (thin line) sinus beat following burst pacing. H MAPD_90_ was longer during the first post-pause beat in R420Q compared to WT hearts and also **I** when normalized to the steady state MAPD_90_ in the same heart. *N* = 10 WT and 9 R420Q hearts. (E,H,I) Unpaired t-test.

We used a transgenic mouse model with a heterozygous RyR2-R420Q mutation (R420Q) to explore the impact of this mutation on repolarization. Left ventricular epicardial monophasic action potentials (MAP) and pseudo-ECGs were recorded in isolated WT and R420Q mouse hearts. Fig. 2A shows an exemplar recording from an R420Q heart in which a ventricular ectopic beat (VEB) occurred during AP repolarization of the first sinus beat following a pause in 10 Hz pacing. This is seen more clearly on an expanded timescale in Fig. 2B, where it is overlaid with a normal beat to emphasise that the ectopic beat occurred near the peak of the corresponding ECG T-wave. VEB occurred during AP repolarization following a pause in 3/9 R420Q hearts (Fig. 2C), but not in any WT hearts (*N* = 10). The mean pause interval between final 10 Hz stimulus and first sinus beat was 364 ± 4 ms in WT and 408 ± 35 ms in R420Q hearts, which was not significantly different (*P* = 0.44; unpaired *t*-test). The mean cycle length during sinus rhythm following this stimulation protocol was 245 ± 23 ms in WT and 346 ± 51 ms which was also not significantly different (*P* = 0.074; Mann-Whitney test). In some hearts, subthreshold diastolic depolarizations in the MAP were observed during the pause that might indicate delayed afterdepolarizations (Fig. S2), however these did not initiate ectopic beats or VT/VF in any R420Q hearts under these experimental conditions.

We next investigated whether AP prolongation could account for triggered activity during repolarization. The MAP duration at 90 % repolarization (MAPD_90_) and average cycle length (WT, 302 ± 20 ms; R420Q, 327 ± 31 ms; *P* = 0.56 unpaired t-test) were not different between WT and R420Q hearts during steady sinus rhythm (Fig. 2D,E). It was reported that pauses in rhythm caused a transient increase in U-wave amplitude in R420Q patients, [13] and similarly we found increased post-extrasystolic U-waves in an RyR2-C2277R patient (Supp. Fig. 1B), so we examined whether pauses in rhythm could induce AP lengthening in R420Q mouse hearts. A pacing protocol with a brief burst of 10 stimuli at short cycle length (28–60 ms) was used to elicit maximal heart rate (WT, 12.6 ± 0.4 Hz; R420Q, 12.5 ± 0.9 Hz; *P* = 0.92 unpaired t-test) then pacing was switched off (Fig. 2F). Fig. 2G shows exemplar MAP recordings during the first and fifth sinus beat in a WT and R420Q heart following pacing. The MAPD_90_ of the first sinus beat was longer in R420Q compared to WT hearts (Fig. 2H) including when post-pause MAPD_90_ values were normalized by the steady state MAPD_90_ in each heart (Fig. 2I).

### 3.2 Beta-adrenergic activation increases susceptibility to premature stimulation

**Fig. 3:**
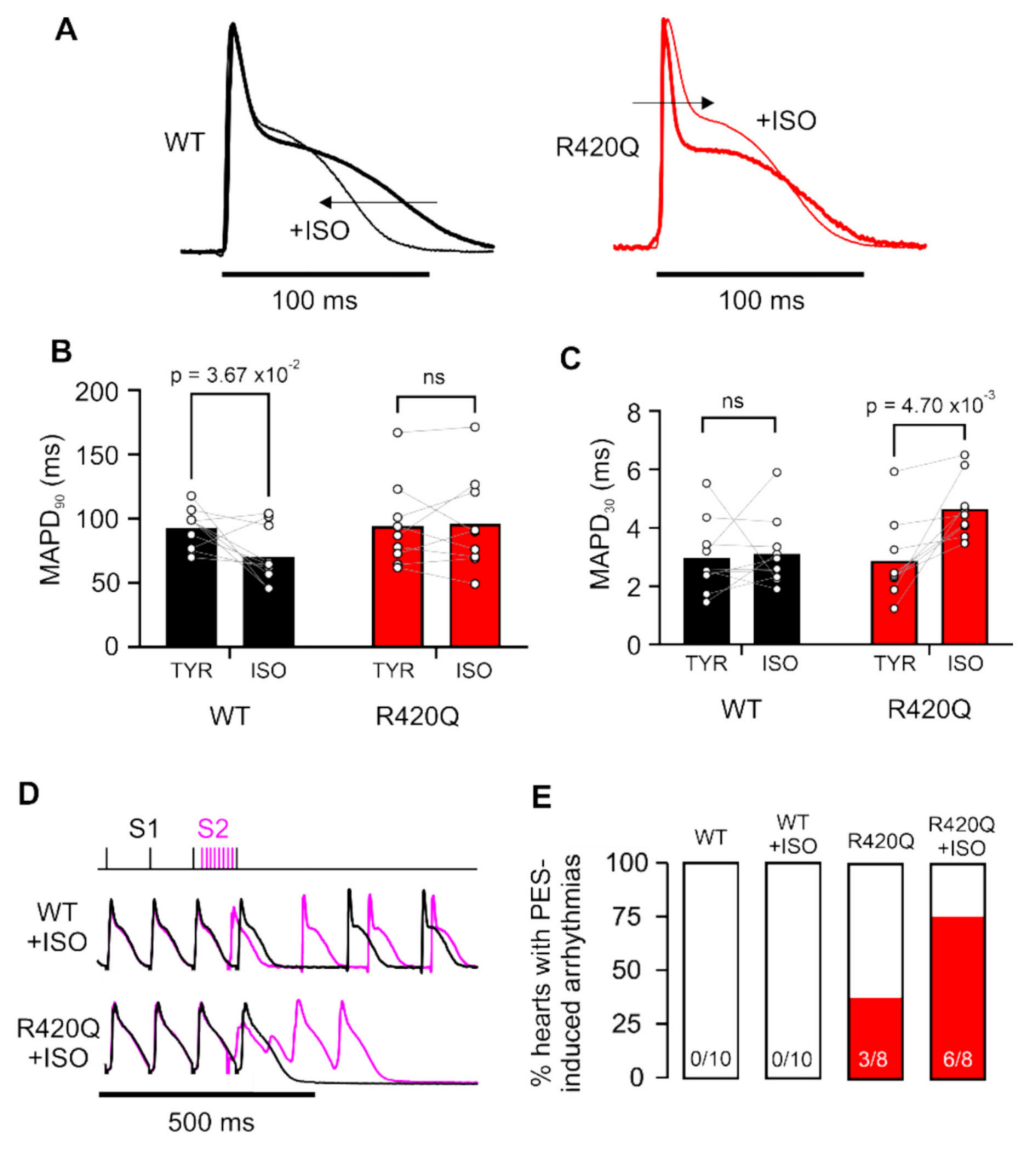
Altered repolarization in response to isoproterenol and premature stimulation in R420Q mouse hearts. A Exemplar MAP recordings from a WT and R420Q heart in control conditions (thick lines) and during stimulation with isoproterenol (100 nmol/L; ISO) (thin lines). In these examples, the WT MAPD at 90 % repolarization (MAPD_90_) was 98.4 ms in TYR and 66.0 ms in ISO, and the R420Q MAPD_90_ was 77.8 ms in TYR and 70.4 ms in ISO. B ISO shortened mean MAPD_90_ in WT but not R420Q hearts. C Conversely, ISO increased MAPD at 30 % repolarization in R420Q but not WT hearts. D S1S2 protocol involving progressively shorter S2 intervals (top) imposed at the end of steady pacing at 10 Hz. Exemplar recordings from a WT and R420Q heart in ISO when the final stimulus is at the normal 100 ms S1 cycle length (black lines) and at a shorter S2 interval of 80 ms (magenta lines). In the WT heart the shorter S2 elicits a single AP, whereas in the R420Q heart the same S2 pulse triggers EAD-like behaviour. Only a single S2 recording is shown for clarity but all hearts were subjected to all S2 intervals. E Percentage of hearts that developed arrhythmias in response to premature S2 electrical stimuli (PES) in control and ISO solutions (N = 10 WT and *N* = 8 R420Q hearts). (B,C) Paired t-test. (For interpretation of the references to colour in this figure legend, the reader is referred to the web version of this article.)

When hearts were exposed to 100 nmol/L isoproterenol (ISO) the sinus cycle length shortened similarly in both WT and R420Q hearts (WT, 212 ± 31 ms; R420Q, 260 ± 40 ms; *P* = 0.43, unpaired t-test) and differential effects on repolarization between WT and R420Q hearts emerged. As might be expected, ISO shortened MAPD_90_ and slightly increased phase 2 of the AP in WT, but in R420Q hearts the increase in phase 2 became larger with almost no effect on MAPD_90_ (Fig. 3A,B). The change in phase 2 resulted in the MAPD at 30 % repolarization increasing in R420Q hearts but not in WT hearts (Fig. 3C). These data are consistent with reduced repolarization reserve in R420Q hearts, caused by imbalance between inward and outward currents, [28] which could increase vulnerability to extrasystoles. [29] We therefore investigated whether R420Q hearts were more susceptible to arrhythmias triggered by premature electrical stimulation (S1S2 protocol) (Fig. 3D). Premature S2 stimuli induced runs of triggered activity in 3/8 R420Q hearts in normal Tyrode’s solution (TYR), and in 6/8 hearts in the presence of ISO (Fig. 3E), whereas this did not occur in any WT hearts (Fig. 3E). Optical mapping was used in some additional experiments to monitor membrane potential changes in the mouse left ventricle epicardium during S1S2 stimulation. Fig. S3 shows that premature S2 stimulation delivered during repolarization in an R420Q heart prevented repolarization in tissue nearest the electrode (without eliciting a full AP) but that this depolarized region appeared to trigger activity in the surrounding area resulting in brief tachycardia. We observed that premature ectopic activity was also associated with an increase in U-wave amplitude following premature beats in an R420Q patient (Fig. S4).

### 3.3 Early afterdepolarizations in R420Q ventricular myocytes

**Fig. 4:**
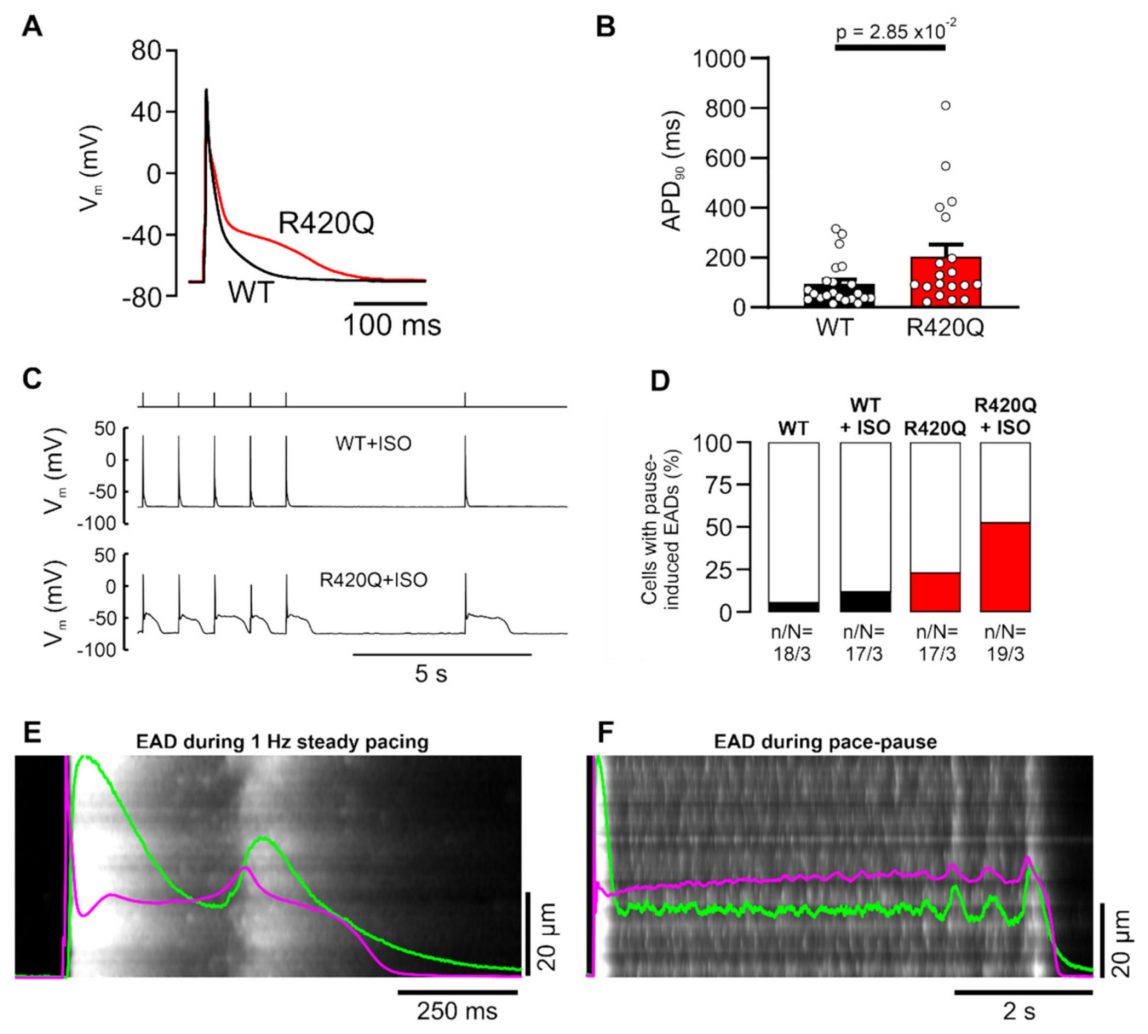
Increased prevalence of EADs and APD lengthening in isolated R420Q mouse ventricular myocytes. A Exemplar APs recorded in isolated WT and R420Q ventricular myocytes at 22 °C using whole-cell patch clamp. B APD_90_ was longer in R420Q myocytes during pacing at 1 Hz in normal Tyrode’s solution. C Exemplar WT and R420Q APs during a pace (1 Hz) pause (5 s) protocol (top panel) in the presence of ISO. Some R420Q cells developed EADs during steady pacing, and especially in the beat following the pause. D Proportion of WT and R420Q cells that developed EADs during a pacing-pause protocol in normal Tyrode’s and during ISO stimulation. E Exemplar confocal Ca^2+^ line scan recording of an EAD during steady pacing and F following the pace-pause protocol illustrated in (C) in an R420Q myocyte during ISO stimulation. Large numbers of spontaneous late Ca^2+^ sparks are visible coinciding with delayed recovery of the cellular average Ca^2+^ transient (green line) and membrane potential (magenta) shown normalized as an overlay. Note the different time scales in panels E & F. (B) n/N (cells/hearts) = 19/3 WT and 22/5 R420Q. (D) n/N (cells/hearts) as indicated below bars. (B) Unpaired t-test. (For interpretation of the references to colour in this figure legend, the reader is referred to the web version of this article.)

The APD_90_ of single ventricular myocytes isolated from R420Q hearts was longer than WT cells during steady pacing at 1 Hz at 22 °C, as illustrated in Fig. 4A and summarized in Fig. 4B. In some R420Q cells, application of ISO (100 nmol/L) produced spontaneous EADs during 1 Hz pacing. To quantify the prevalence of EADs we used a pacing-pause protocol (illustrated in Fig. 4C). Few WT cells developed EADs following the pause in either TYR or ISO, whereas ~50 % of R420Q cells exhibited EADs following the pause in ISO (Fig. 4D). These experiments were repeated at a physiological temperature (35 °C) and similar results were found (Fig. S5). The Ca^2+^ current plays a role in EAD genesis, [30] however I_Ca_ density was similar in R420Q and WT cells (Fig. S6 and [22]) suggesting that I_Ca_ was not responsible for the difference in EADs. Increased Ca^2+^ leak during systole, in the form of ‘late Ca^2+^ sparks’ (LCS), can impair repolarization reserve in heart failure, resulting in AP lengthening and EADs by increasing inward NCX current. [31] LCS are Ca^2+^ sparks that occur during the Ca^2+^ transient decline after the initial AP-evoked Ca^2+^ release, [32] however their role in other diseases, including CPVT, remains unknown. Exemplar confocal Ca^2+^ line scan recordings of EADs during steady 1 Hz pacing (Fig. 4E) and following the pause (Fig. 4F) showed large numbers of LCS occurring in R420Q cells stimulated with ISO. In the latter case, V_m_ was initially in a quasi-stable state coinciding with dyssynchronous Ca^2+^ release, which eventually became more coordinated and in-phase with V_m_ leading to full repolarization. [33]

### 3.4 Increased frequency of late Ca^2+^ sparks in R420Q myocytes

**Fig. 5:**
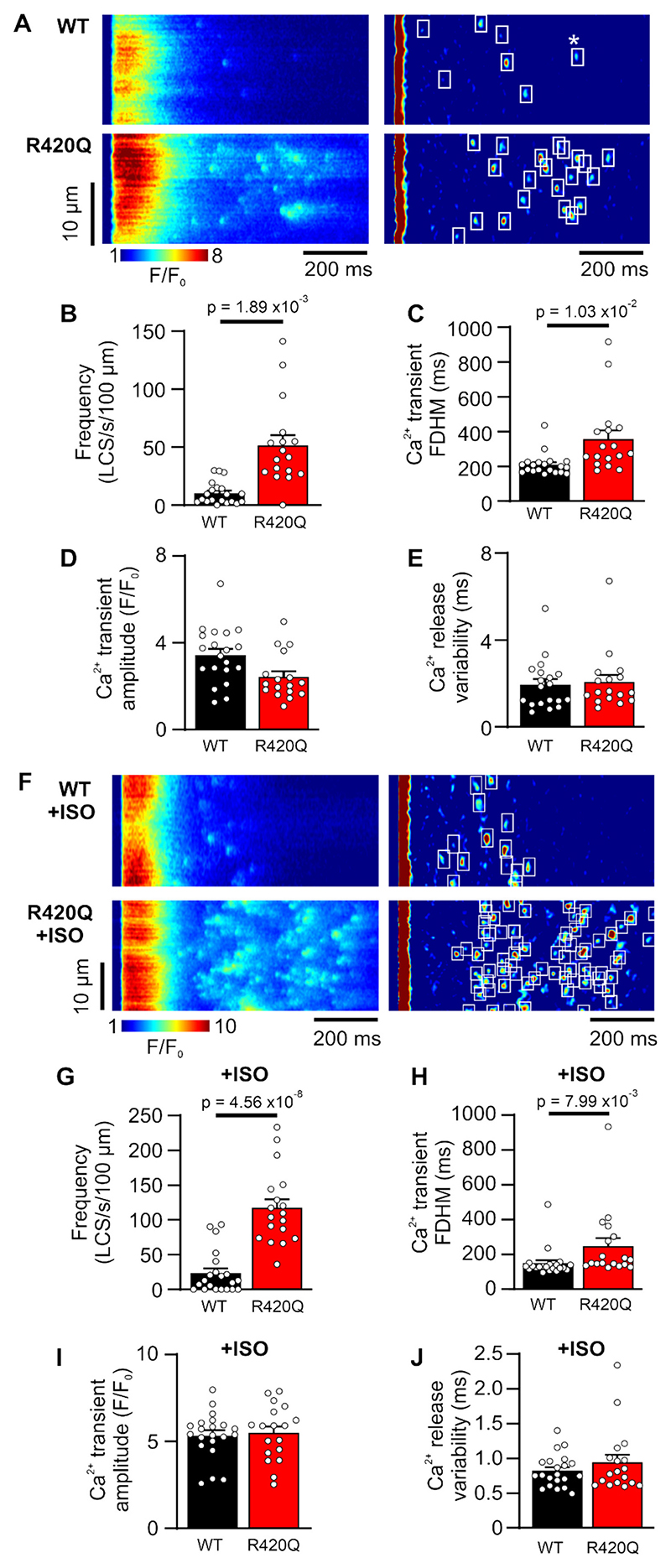
Increased frequency of late Ca^2+^ sparks (LCS) in isolated R420Q mouse ventricular myocytes. A Exemplar confocal Ca^2+^ line scan recordings. LCS during the Ca^2+^ transient decay were detected by automated algorithm in high-pass filtered recordings (white boxes, right panels). Event marked * is a diastolic Ca^2+^ spark occurring after [Ca^2+^] has returned to within 10 % of the resting level. B LCS frequency and C the full-duration at half maximum (FDHM) of the Ca^2+^ transient were increased in R420Q cells. D Ca^2+^ transient amplitude and E electrically-evoked Ca^2+^ release synchrony were similar between groups. F Increased LCS frequency during isoproterenol (ISO) stimulation. G LCS frequency and H Ca^2+^ transient duration was greater in R420Q myocytes during ISO stimulation, whereas I Ca^2+^ transient amplitude and **J** Ca^2+^ release synchrony were not different. (B-E) n/*N* = 20/4 WT and 17/6 R420Q cells/hearts, (G-J) n/*N* = 21/4 WT and 18/5 R420Q cells/hearts. Data points represent the mean value from each cell. Nested unpaired t-test.

Fig. 5A shows exemplar confocal Ca^2+^ line scan recordings of Ca^2+^ transients in a WT and R420Q myocyte in normal Tyrode’s solution. A small number of LCS occurred during the Ca^2+^ transient decay in WT, but were more frequent in R420Q cells, as can be seen more easily after high-pass image processing and automated LCS detection (white boxes, right panels). The increased LCS frequency in R420Q cells (Fig. 5B) was associated with a longer Ca^2+^ transient duration compared to WT cells (Fig. 5C). LCS can be promoted by impaired early Ca^2+^ release and increased SR load, [31,32,34] but the Ca^2+^ transient amplitude and synchrony of electrically evoked Ca^2+^ release was not different in R420Q cells (Fig. 5D and Fig. 5E, respectively). In addition, immuno-cytochemistry revealed that the transverse-tubule network was not disrupted in R420Q cells meaning that orphaning of RyR2 clusters was not involved, [35] as there was similar regularity and colocalization between RyR2 and the sarcolemma marker caveolin-3 (Fig. S7). SR Ca^2+^ content was significantly reduced in R420Q compared to WT cells in TYR and there was a trend for a reduction in ISO (Fig. S8), assessed by integration of the NCX current after rapid application of caffeine under voltage clamp, [36] consistent with a previous report. [22] Reduced SR Ca^2+^ load would normally be expected to reduce the probability of Ca^2+^ sparks occurring. [37]

ISO (100 nmol/L) increased LCS frequency in WT cells from 10.4 ± 2.2 to 23.6 ± 6.7 LCS/s/100 μm, but dramatically increased LCS frequency in R420Q cells to 117.3 ± 12.6 LCS/s/100 μm compared to 51.6 ± 8.8 LCS/s/100 μm in Tyrode’s (Fig. 5F,G). This difference was underscored by the fact that whereas LCS were not detectable in ~30 % of WT cells during ISO stimulation, LCS were detected in all R420Q cells in ISO (*P* < 0.001 vs WT ISO; χ^2^ test).

We used dual Ca^2+^/V_m_ cardiac optical mapping in WT and R420Q hearts to establish whether the Ca^2+^ transient was also prolonged in intact hearts. External Ca^2+^ was increased to 1.8 mmol/L during these experiments, which was found to increase the basal heart rate to 6.1 ± 0.3 Hz in WT and 6.0 ± 0.4 in CPVT hearts before dye loading (*P* = 0.84 WT vs R420Q unpaired *t*-test; *N* = 4 WT and *N* = 3 R420Q hearts). The mean Ca^2+^ transient duration was indeed significantly prolonged in R420Q hearts during normal sinus rhythm during stimulation with 1 μmol/L ISO (Fig. S9).

### 3.5 Late Ca^2+^ sparks prolong action potential duration in R420Q myocytes

**Fig. 6:**
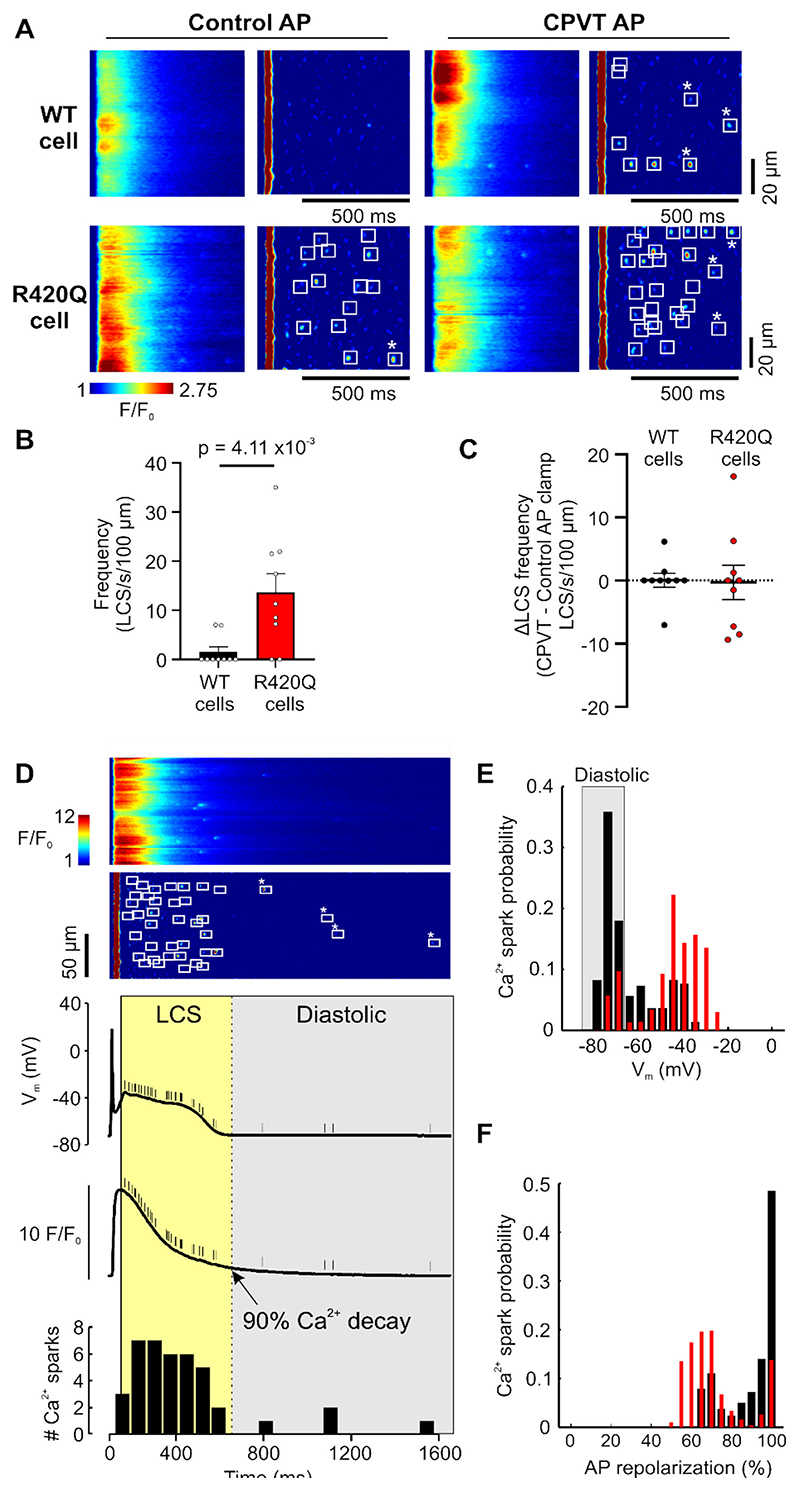
Time and voltage dependence of LCS in WT and R420Q mouse ventricular myocytes. The WT and R420Q exemplar APs from Fig. 4A were used as canonical Control and CPVT command waveforms for AP voltage clamp experiments, respectively. A Exemplar confocal Ca^2+^ line scan recordings in a WT (top panels) and R420Q cell (bottom panels) under voltage clamp with a Control (left panels) or CPVT AP (right panels) following standardized Ca^2+^ loading with a sequence of square pulses to +10 mV (see Supplementary Materials). B LCS frequency was greater in R420Q compared to WT cells under voltage clamp with a Control AP. C The frequency of LCS was not dependent on the particular AP waveform in either WT or R420Q cells (shown as the difference in LCS frequency clamped with a CPVT AP minus frequency with a Control AP). D (from top to bottom) Exemplar Ca^2+^ line scan recording, detected Ca^2+^ sparks, membrane potential, cellular average Ca^2+^ transient, and histogram of detected Ca^2+^ sparks in an R420Q cell under current clamp. Ca^2+^ spark events are marked by short lines on V_m_ and Ca^2+^ transient plots. Events between the peak and 90 % recovery of Ca^2+^ transient were included in LCS analysis, and later events classed as diastolic and analysed separately (indicated by * in panels A&D). E & F Probability distribution of where LCS occurred in current clamped cells in relation to E V_m_ and F as a % of AP repolarization, constructed from Ca^2+^ sparks detected in n/*N* = 16/ WT and n/*N* = 9/2 R420Q cells/hearts. (B–C) n/N (cells/hearts) = 9/3 WT and 9/4 R420Q. Mann-Whitney test.

To establish whether differences in AP shape and/or duration caused the increase in LCS frequency or alternatively, were a result of the prolonged Ca^2+^ transient and increased LCS, we used the AP voltage clamp (AP clamp) technique with either a typical WT (control AP) or R420Q AP (CPVT AP) as the voltage command. Fig. 6A shows exemplar confocal Ca^2+^ line scans recorded under AP clamp following standardized SR Ca^2+^ loading by a series of square voltage steps to +10 mV. Overall, LCS frequency was greater in R420Q cells compared to WT cells when clamped with a control AP (Fig. 6B), but within each cell type LCS frequency did not change depending on which AP waveform was used (Fig. 6C).

In AP clamp experiments, some Ca^2+^ sparks occurred after V_m_ and [Ca^2+^] had returned nearly to resting levels and therefore would not normally contribute to APD lengthening but might contribute to DADs (asterisks in Fig. 6A). We performed temporal analysis of LCS in current clamped myocytes, which preserves the normal Ca^2+^ and voltage feedback mechanism (Fig. 6D). To increase the number of LCS for analysis the first beat after a 5 s pause in 1 Hz pacing with ISO was analysed. Most Ca^2+^ sparks occurred in R420Q cells between around −45 and − 30 mV, corresponding to approximately 55–70 % AP repolarization, whereas in WT cells a smaller proportion of Ca^2+^ sparks occurred around −40 mV, with most occurring nearer the resting membrane potential (WT, −74.3 ± 0.8 mV; R420Q, −74.5 ± 0.7 mV; *P* = 0.80) and after repolarization was complete (Fig. 6E,F). LCS also occurred during the AP in R420Q cells at 35 °C, albeit with more difficultly detecting LCS due to the much faster Ca^2+^ cycling at this temperature (Fig. S5E).

### 3.6 Reduced refractoriness and increased sensitivity of RyR2 promote LCS

**Fig. 7:**
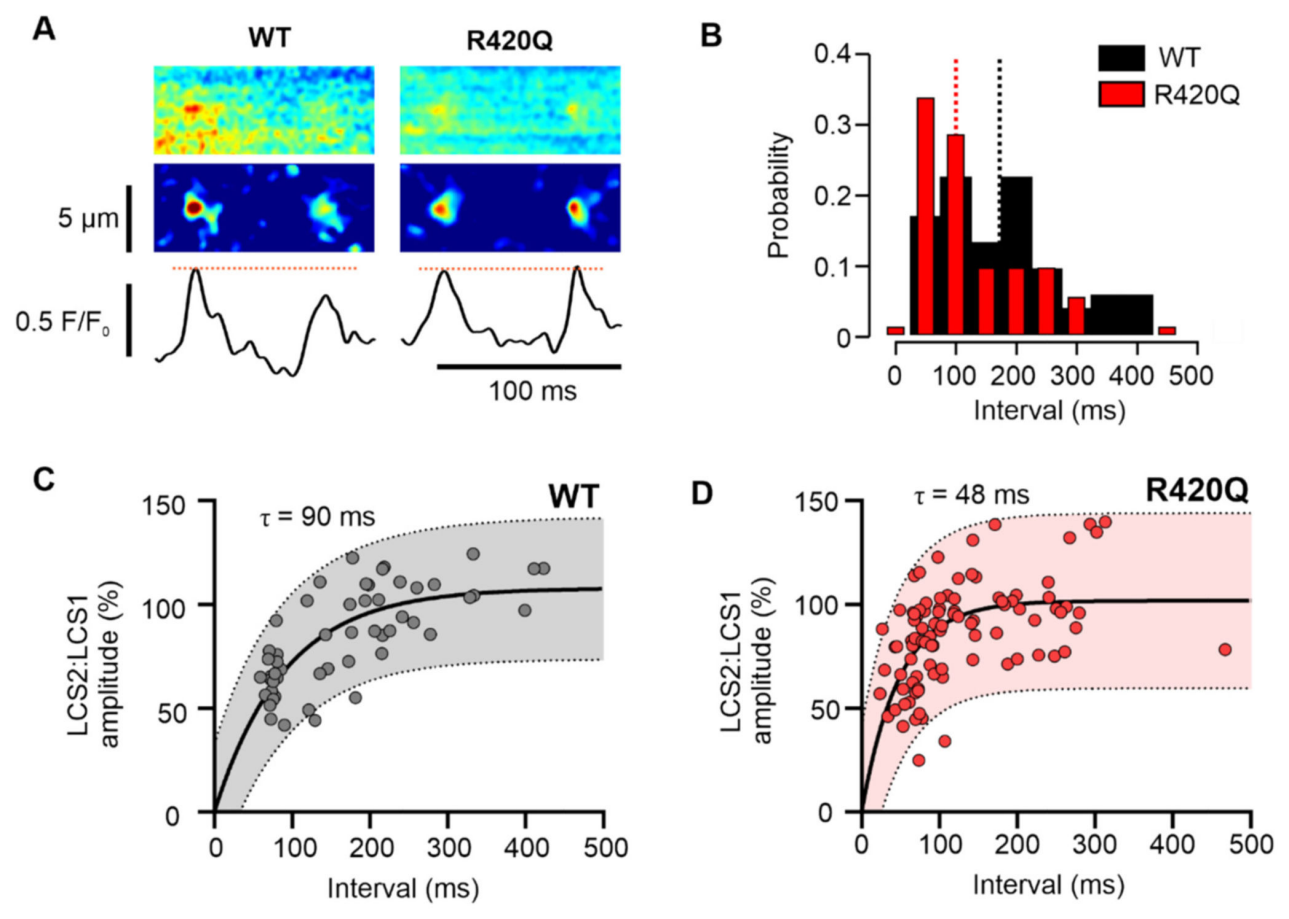
Reduced Ca^2+^ release refractoriness increases LCS probability in R420Q mouse ventricular myocytes. A Exemplar line scan recordings showing pairs of LCS occurring from the same location ~70 ms apart. Middle panels show high-pass filtered recordings. Lower panels show the mean fluorescence through the centre of LCS. B Probability density of intervals between LCS pairs. Dashed lines show the median inter-LCS interval for WT (172 ms) and R420Q cells (99 ms). C Recovery of the second LCS (LCS2) relative to the first (LCS1) was slower in WT compared to D R420Q cells. (C,D) Data points represent one pair of LCS. Shaded areas indicate 95 % prediction interval. Solid lines show a single exponential fit to the data. (B,C,D) 53 LCS pairs from 6/2 WT cells/hearts and 94 LCS pairs from 3/3 R420Q myocytes/hearts.

The increased LCS frequency in R420Q cells might be explained by a change in the refractory period for Ca^2+^ sparks. It is known that Ca^2+^ release sites can fire more than once during the Ca^2+^ transient decay. [32] This can result in multiple LCS at release sites, each separated by a refractory period, that is (at least partly) due to the delay in junctional SR refilling. [32,38] Fig. 7A shows exemplar recordings from WT and R420Q myocytes where an initial LCS was followed by a second LCS ~70 ms later (top and middle panels). Fig. 7B shows that the probability density distribution for the interval between LCS was skewed towards shorter intervals, with the median inter-LCS interval reduced from 172 ms in WT cells to 99 ms in R420Q cells (*P* = 0.006; Mann Whitney unpaired t-test). The bottom panel of Fig. 7A shows the intensity profile through the centre of each LCS pair. In WT cells the amplitude of the second LCS recovered with a time constant of 90 ms (Fig. 7C), whereas the recovery in R420Q cells was ~2× faster (Fig. 7D). Such an effect would not only increase the frequency of LCS in R420Q but also their individual contribution to the slowing of the Ca^2+^ transient decay.

Most RyR2 mutations associated with CPVT are gain-of-function, [4] however some mutations have atypical phenotypes. [39] Diastolic Ca^2+^ spark frequency was greater in R420Q myocytes (Fig. S10) confirming that R420Q is a gain-of-function CPVT mutation. We next investigated whether our observations might extend to RyR2 hyperactivity more generally by using an in vitro model of CPVT (low-dose caffeine plus isoproterenol). [40] Fig. S11 shows exemplar confocal Ca^2+^ line scan recordings of WT cells in the presence of 1 μmol/L ISO and ISO + 1 mmol/L caffeine. [41] LCS frequency was greatly increased and the Ca^2+^ transient duration was prolonged in the presence of caffeine (Fig. S11C, D). Furthermore, premature electrical stimulation did not elicit arrhythmias in WT hearts in the presence of 100 nmol/L ISO alone (Fig. 3E), but arrhythmias did occur when caffeine was administered in combination with ISO (Fig. S12).

### 3.7 Computer simulations of hyperactive RyR2 in CPVT

**Fig. 8:**
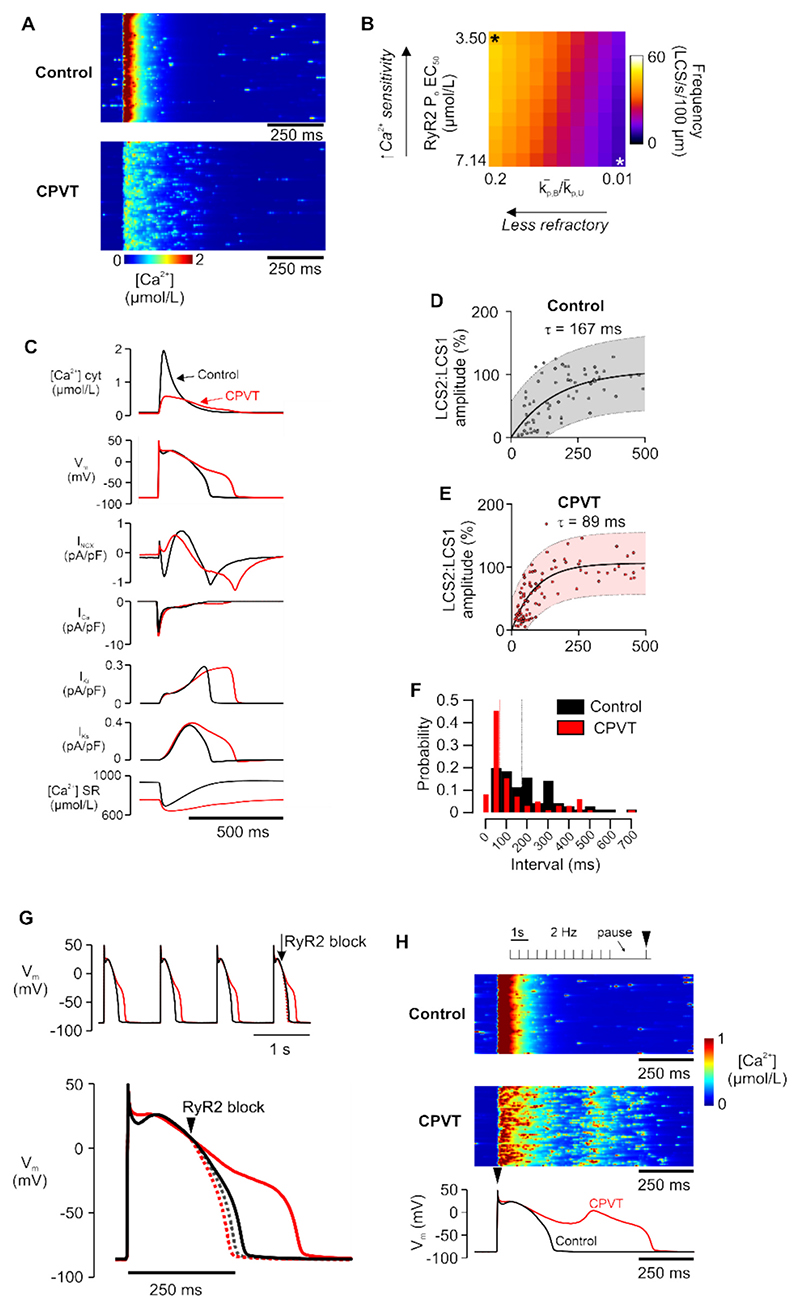
Hyperactive RyR2 induces CPVT-like aberrant Ca^2+^ dynamics in a computer model of spatially distributed Ca^2+^ release with rabbit electrophysiology. [42] A Simulated Ca^2+^ line scans with normal (Control) and hyperactive CPVT RyR2 properties B (increased Ca^2+^ sensitivity and reduced refractoriness). B Heatmap showing interaction between RyR2 Ca^2+^ sensitivity and refractoriness on LCS frequency. White and black asterisks indicate values used for Control and CPVT RyR2 simulations, respectively. C Ca^2+^ transient amplitude was decreased and the Ca^2+^ transient and AP duration was increased in CPVT (red lines) compared to Control (black lines). Inward I_NCX_ and late I_Ca_ were increased during repolarization in CPVT. D & E Amplitude recovery of pairs of LCS during simulated voltage clamp at −40 mV. F Probability density of intervals between LCS pairs, with the median interval in Control (167 ms) and CPVT (89 ms) indicated. G (upper panel) Control and CPVT simulations were paced to steady state at 1 Hz then SR Ca^2+^ release was blocked at the time indicated. (lower panel) Expanded time view of the final AP during RyR2 block (dashed lines). Also see Fig. S13. H A pace-pause protocol induced EADs in CPVT but not in Control simulations. (For interpretation of the references to colour in this figure legend, the reader is referred to the web version of this article.)

Finally, we sought to extend our main experimental findings with the R420Q mutation in mouse to predict how RyR2 hyperactivity might behave in a larger species with different ion channel contribution and AP morphology. For this we used an established computer model of a ventricular myocyte with three-dimensional spatially distributed Ca^2+^ release that reproduces the LCS phenomenon and uses rabbit electrophysiology (see also Fig. S14). [31,42] CPVT RyR2 properties were simulated by increasing RyR2 Ca^2+^ sensitivity ~2× and reducing RyR2 refractoriness (see Supplementary Materials). The simulated Ca^2+^ line scans in Fig. 8A show that many more LCS occurred in CPVT simulations compared to normal RyR2 properties in the presence of ISO, and this was dependent on RyR2 sensitivity and refractoriness (Fig. 8B). The LCS rate with normal RyR2 was 8.9 LCS/s/100 μm and with CPVT RyR2 was 43.9 LCS/s/100 μm. Notably, this ~5× difference in LCS rate in simulations was close to the ~5× difference in LCS frequency between WT (23.6 LCS/s/100 μm) and R420Q myocytes (117.3 LCS/s/100 μm) measured experimentally during ISO stimulation (c.f. Fig. 5G). CPVT RyR2 reduced the Ca^2+^ transient amplitude but increased its duration and prolonged the AP (Fig. 8C). Steady state SR load decreased by ~30 % from 896 to 636 μmol/L in CPVT, similar to the ~30 % decrease in SR load measured in TYR and ISO experimentally (Fig. S8).

At 50 % AP repolarization, corresponding to −18 mV and approximately mid-phase 3 repolarization, I_NCX_ was ~4× greater in CPVT (−0.58 pA/pF) compared to control (−0.14 pA/pF). The increased inward current effectively negated the combined outward current from I_Ks_ and I_Kr_ at the same timepoint (0.50 pA/pF), whereas in Control there was an excess of these outward currents by around 0.33 pA/pF (Fig. 8C). Peak I_Ca_ (7.0 pA/pF Control vs 8.0 pA/pF CPVT) and I_Ca_ at 50 % repolarization (−0.48 pA/pF Control vs −0.40 pA/pF CPVT) were similar magnitude between simulations, although late I_Ca_ persisted for longer in CPVT possibly due to reduced Ca^2+^-dependent inactivation.

Increased late I_Ca_ or I_NCX_ could feasibly explain APD lengthening, so we performed further simulations where SR Ca^2+^ release was blocked midway through AP repolarization. Fig. 8G shows that blocking RyR2 after the initial evoked Ca^2+^ release almost normalized the prolonged APD in CPVT. This approach dramatically suppressed LCS in CPVT, but had a smaller effect in Control simulations (Fig. S13A). The magnitude and duration of inward I_NCX_ was reduced and there was a brief increase in reverse-mode outward I_NCX_ at the moment of block due to decreased subsarcolemmal [Ca^2+^] (Fig. S13B). Imposing the original I_Ca_ from a normal CPVT simulation did not fully restore APD when SR Ca^2+^ release was blocked (Fig. S13B), suggesting I_NCX_ was critical in APD lengthening. Finally, a simulated pacing-pause protocol induced EADs in CPVT but not control simulation (Fig. 8D). Thus, hyperactive RyR2 can contribute to increased LCS and inward I_NCX_ that could reduce repolarization reserve and enable arrhythmias arising during repolarization in CPVT.

## 4 Discussion

### 4.1 Early afterdepolarization involvement in CPVT caused by gain-of-function RyR2-R420Q

The origin of arrhythmias and extrasystoles in CPVT is usually attributed to diastolic Ca^2+^ waves and DADs with less attention directed towards ventricular repolarization as a potential source. [5–8] It was proposed that CPVT patients may be susceptible to both DADs and EADs, [11,43] although an EAD arrhythmia mechanism has not been shown in patients. Our preclinical investigation shows that increased Ca^2+^ leak during systole, caused by hyperactive RyR2, results in AP prolongation and EADs that could contribute towards the inducibility of arrhythmias in CPVT. This does not require SR Ca^2+^ overload and thus is mechanistically distinct from ‘Ca^2+^-release deficiency syndrome’ caused by hypoactive RyR2. [39] Nonetheless, the LCS-EAD mechanism is likely to be exacerbated by increased SR load and this could explain pause-induced abnormal repolarization in CPVT (due to an increase in SR filling time) that is separate from the well-established DAD-mediated mechanism.

We found that APD lengthening in R420Q mouse hearts mainly occurred following pauses and during beta-adrenergic activation, but not during steady rhythm (Fig. 2). This would be consistent with clinical findings that QT interval in CPVT patients typically falls within a normal range at rest, [44,45] but that in some cases QT lengthening and augmented U-waves can occur following pauses, [14,24] or when provoked by adrenergic agents. [46,47] Prolonged resting QT intervals or notched T-waves have been reported in some CPVT patients, [11,12,48,49] although this was not the case in our R420Q patients that instead showed pronounced U-waves (Table 1).

More than 200 mutations in RyR2 have been associated with CPVT, [4] so there may be diversity in phenotype or mutation-specific effects, which raises the question of whether our results are unique to the R420Q mutation? Although it is not feasible to confirm whether the LCS-EAD mechanism occurs in all cases, we did find a similar increase in systolic Ca^2+^ leak following acute RyR2 sensitization in an in vitro model of CPVT and in computer simulations that should mimic the broader phenomenon of RyR2 gain-of-function. Additionally, patterns of abnormal systolic Ca^2+^ release were reported in the CASQ2-R33Q model of CPVT [50] and in CaMKII_δC_ overexpressing mice [51] that would be consistent with increased LCS resulting from RyR2 hyperactivity. It is possible that the clinical presentation may not be definitive as to the underlying arrhythmia mechanism and that there is likely to be some overlap between different inherited heart conditions. In connection with this, a knock-in KCNJ2-R67Q mouse model of Andersen-Tawil Syndrome expressing a loss-of-function Kir2.1 channel with catecholamine-induced decrease in I_K1_ density developed CPVT-like bidirectional and polymorphic ventricular tachycardia that was likely initiated by EADs without QT lengthening, rather than DADs. [23]

### 4.2 Prolonged repolarization in CPVT mediated by late Ca^2+^ sparks

The increase in LCS and slowing of Ca^2+^ transient decay in R420Q cells was especially pronounced during beta-adrenergic stimulation. The concept of “repolarization reserve” refers to the normal redundancy of repolarizing K+ currents, however drugs or disease that decrease outward or increase inward currents can alter this balance resulting in alterations or failure of repolarization. [28,29] It is known that Ca^2+^ leak during repolarization can drive afterdepolarizations in other disease contexts, such as heart failure and the long QT syndrome type 2. [31,42] Inclusion of the Ca^2+^ buffer BAPTA in the patch pipette disrupted the normal feedback between intracellular Ca^2+^ and V_m_ via I_NCX_ and shortened APD and suppressed triggered activity during beta-adrenergic stimulation in WT and R420Q cells. [22] APD shortening is a normal physiological response to beta-adrenergic stimulation and increased heart rate, whereas the lack of APD shortening in R420Q hearts may increase the vulnerability to re-entrant circuits forming due to functional conduction block and extrasystoles originating from regional heterogeneity in repolarization.

Increased RyR2 sensitivity should cause more efficient evoked Ca^2+^ release and therefore greater SR Ca^2+^ depletion, [22] that might be expected to *decrease* LCS frequency, [34] but this will be offset by the shorter coupling interval and faster recovery between LCS. Recovery of Ca^2+^ spark amplitude is influenced by the rate of junctional SR refilling [38] but occurs on a slightly faster time course due to, at least in part, the steep dependence of RyR2 open probability on dyadic [Ca^2+^]. [52] Beta-adrenergic stimulation will accelerate SR refilling and thus further enhance the re-triggerability of LCS. [53] Faster Ca^2+^ cycling at physiological temperatures should also increase the recovery of LCS due to increased SERCA2a activity and indeed LCS were observed during the AP at 35 °C in CPVT myocytes (Fig. S5E). The time course of the Ca^2+^ transient and AP that we observed using dual Ca^2+^/V_m_ optical mapping (Fig. S9) means that it is feasible that LCS could contribute to AP lengthening during normal heart function, and especially with hyperactive RyR2 due to the shorter LCS refractory period shifting more Ca^2+^ release within the time frame of AP recovery, although this remains to be demonstrated.

### 4.3 Synchronization of spontaneous Ca^2+^ release by heart rhythm

A large number of cells must develop DADs or EADs in near unison (~700,000 in ventricular tissue, but possibly fewer in specialised regions such as the His-Purkinje fibre/endocardium junction [54]) to overcome the source-sink mismatch caused by electrotonic coupling, for triggered activity to propagate. [55] Additionally, the membrane input impedance in diastole is lower than during repolarization, so that I_NCX_ will become more effective at causing depolarization (altering dV/dt) during the early phase of the AP than during diastole. Therefore, a smaller LCS-mediated I_NCX_ (~0.2 pA/pF) can slow or reverse AP repolarization and facilitate I_Ca_ and/or I_Na_ reactivation during EADs as a source of triggered beats. [31] It is possible that the influence of heart rhythm on Ca^2+^ cycling and AP duration may also serve to entrain otherwise stochastic LCS across sufficient cells to further increase the probability of ectopic beats.

Premature stimulation triggered EAD-like behaviour in R420Q hearts (Fig. 3D,E) and premature beats were associated with increased U-wave amplitude in R420Q patients (Fig. S4). Premature stimulation can modulate repolarization gradients across the heart and give rise to conduction block that becomes a substrate for reentry, [56] and this may be exacerbated by increased spontaneous Ca^2+^ release delaying and causing nonuniformities in repolarization. [40] Increased repolarization dispersion could contribute to U-waves, as shown by the formation of TU complexes due to delayed phase 3 repolarization in canine left ventricular wedge preparations during hypokalemia. [18] At the cellular level, premature APs will elicit smaller Ca^2+^ transients, due to incomplete SR refilling, and less Ca^2+^-dependent inactivation of I_Ca_ causing a net increase in Ca^2+^ entry and SR load for subsequent beats that will increase the likelihood of both DADs and EADs.

### 4.4 Interaction with repolarization mechanisms in other species

The principal repolarization mechanisms in mouse ventricle are different from larger species, such as rabbit and human, with greater reliance on I_to_ and little or no I_Kr_ and I_Ks_, whereas I_Kr_ and I_Ks_ are major determinants of repolarization in humans, especially upon beta-adrenergic activation. The shorter plateau phase of the rodent AP means that a larger or longer Ca^2+^ transient will increase I_NCX_ more than in humans, where the more positive plateau potential will oppose inward I_NCX_. Conversely, reduced Ca^2+^-dependent inactivation of I_Ca_ due to smaller Ca^2+^ transient amplitude could prolong APD more in humans. [57] We modified the electrophysiology of the 3D ventricular myocyte computer model to produce a more ‘mouse-like’ AP or a human ventricular AP [58] (Fig. S14). Although these are not intended as a complete representation of mouse electrophysiology, they do serve to illustrate how RyR2 hyperactivity might interact with different AP morphologies. CPVT RyR2 properties increased LCS and Ca^2+^ transient duration, decreased SR load and Ca^2+^ transient amplitude and increased APD in all simulations regardless of species. The first rabbit CRISPR knock-in model of CPVT with the gain-of-function RYR2-V2475F^+/−^ mutation has recently been generated and preliminary reports in that model indicated prolonged QT interval in homozygous rabbits, and reduced Ca^2+^ transient amplitude and reduced SR load in left ventricular cardiomyocytes from heterozygous rabbits. [59,60]

### 4.5 Limitations

We did not inhibit SR Ca^2+^ release or I_NCX_ in R420Q cells because these approaches would perturb Ca^2+^ load [61] and Ca^2+^-dependent inactivation of I_Ca._ [62] Currently, mouse is the only mammalian model of human CPVT mutations, so it was necessary to use this species for the present work. The mouse ventricular AP is shorter and more triangular than human, which reflects differences in the relative contribution of membrane currents to AP repolarization. We expect the mechanisms underlying EAD genesis should be similar to human, as our key findings are supported by in silico experiments using a cardiac myocyte computer model that shares closer similarities to human electrophysiology. Use of human iPSC-derived cardiac myocytes might avoid some of these problems, but the immature structure (e.g. lack of t-tubules), Ca^2+^ handling and electrophysiology could confound examination of altered excitation-contraction coupling. The RyR2 Arg-420 site is a hotspot for different mutations that produce a typical CPVT phenotype, [4] but we cannot exclude the possibility that some effects may be mutationspecific.

## 5 Conclusion

We show that RyR2 hyperactivity in CPVT caused by the RyR2-R420Q mutation increased Ca^2+^ leak in mouse ventricular myocytes during systole due to an excess of LCS, resulting in compromised repolarization reserve and susceptibility to EAD-mediated arrhythmias. This adds to a well-established role for DADs in this disease and indeed, DADs are most likely at fault when the initiating beat is late coupled. [63–65] Since current treatments do not provide complete protection in all CPVT patients, [66] we suggest that the development of agents to offset loss of repolarization reserve may have some clinical utility.

## Supplementary Material

Supplementary data to this article can be found online at https://doi.org/10.1016/j.yjmcc.2025.07.014. Information on the data underpinning this publication, including access details, can be found in the Cardiff University Research Data Repository at https://doi.org/10.17035/cardiff.29661194

Supplementary material

## Figures and Tables

**Table 1:** Summary ECG parameters of human RyR2-R420Q patients and genotype-negative family member controls. QTc was corrected using Bazzett’s formula (QTc=QT/√RR) QaU, interval from start of the QRS complex to the apex of the U-wave. NS, not significant.

		Control (*N* = 5)				R420Q (*N* = 4)				
		Mean		SEM		Mean		SEM		*P* value
RR interval		0.89	±	0.10		1.00	±	0.07		NS (0.42)
QT (s)		0.39	±	0.02		0.41	±	0.03		NS (0.59)
QTc (s)		0.37	±	0.05		0.41	±	0.02		NS (0.73)
QaU interval (s)		0.49	±	0.02		0.55	±	0.03		NS (0.058)
U integral (ms.mV)		1.79	±	0.60		8.44	±	2.86		2.80 × 10^–2^
