# Supplementary material for: Compromised repolarization reserve in a murine model of catecholaminergic polymorphic ventricular tachycardia caused by RyR2-R420Q mutation
Source: J Mol Cell Cardiol. Author manuscript; Available in PMC 2026 Jun 2. (PMC7619069; doi:10.1016/j.yjmcc.2025.07.014)
Supplement: Supplementary material [file EMS213698-supplement-Supplementary_material.pdf]

Supplementary Materials

Detailed Methods

Ethical statement

Animal care, breeding and experiments were carried out with local ethical approval in accordance with UK Home Office and European Parliament Directive 2010/63/EU guidelines on the use of animals in research. Experiments were performed in adult male and female heterozygous RyR2-R420Q knock-in mice (R420Q) and wildtype littermates as controls (WT) on a C57BL/6J background. The generation of R420Q mice was previously described.[1]

ECG U-wave analysis

U-waves were quantified during individual ECG cycles following the method of [2]. ECG signals were digitized at > 4kHz from original recordings and the components comprising the late phase of the T/U complex were fit by minimization of nonlinear least-squares using the Trust-Region algorithm to the function:

$$y_{T+U} = A_T exp\left(\frac{-t}{\tau_T}\right) + A_U exp\left(\frac{-(t-t_{apex})}{\tau_U}\right)\left(1+exp\left(\frac{-(t-t_{apex})}{\tau_U}\right)\right)^{-2}, \quad \text{(Equation 1)}$$

Where A_T and A_U are the amplitude scaling factors, T_T and T_U the time constants for the T- and U-wave components, respectively, and T_{apex} is a time offset for a Gaussian curve. The first term describes the declining phase of the T-wave with a monoexponential decay and the second term superimposes a shifted Gaussian curve to approximate the U-wave. Fitting was performed in MATLAB 2023a (MathWorks, MA). The QaU interval was calculated from the start of the QRS complex to the peak of the U wave.[3]

Perfused heart model

Mice were killed by stunning and cervical dislocation. Hearts were removed and perfused via the aorta with continuously oxygenated modified Tyrode's solution (containing, in mmol/L: 133 NaCl, 5 KCl, 1 NaH_2PO_4, 10 4-(2-hydroxyethyl)-1piperazineethanesulfonic acid (HEPES), 10 glucose, 1 $CaCl_2$, 1 $MgCl_2$, pH 7.4 with NaOH 35 ± 1 °C) on a gravity-fed Langendorff apparatus. Hearts were positioned in a horizonal cradle constructed from polycarbonate and immersed in a temperature-controlled perfusion chamber (TBC-2.2, MappingLab, Oxford, UK). A pair of electrodes made from Ag/AgCl pellets were positioned near the left ventricle apex and in gentle contact with the right atrium to record a pseudo-ECG in a lead II configuration and help stabilise the heart. Monophasic action potentials (MAP) were recorded from the left ventricle apex using a twisted pair of Teflon coated chloridised Ag wires (0.25mm in diameter).[4] MAP signals were amplified with an isolated DC-coupled differential amplifier and lowpass filtered at 1 kHz. Amplified signals were digitized at 5 kHz using a Digidata 1440a (Molecular Devices, CA). Recordings were made in the presence or absence of isoproterenol (ISO, 100 nmol/L). Blebbistatin (10 µmol/L) was included in the perfusion solution to minimise motion artefacts during MAP recording. Electrical stimuli were delivered via a pair of Ag electrodes (MappingLab, Oxford, UK) using an isolated stimulus generator (SD9, Grass Instruments).

Stimulus protocols were programmed using Clampex (Molecular Devices). Premature electrical stimulation was investigated by pacing hearts to steady state at 10 Hz (S1), then an S2 stimulus at a shorter cycle length (from 20-90 ms in 10 ms increments) was delivered. Arrhythmias were defined, in line with the Lambeth conventions for the study of arrhythmias in preclinical models,[5] as 4 or more consecutive peaks in membrane potential that occurred in response to an S2 stimulus delivered during the repolarization phase of the preceding beat. AP lengthening following a pause was investigated by pacing hearts to steady state at 10 Hz, followed by a short burst (10 stimuli total) of pacing at shorter cycle length (28-60 ms) to elicit a maximal heart rate (WT, 12.6 ± 0.4 Hz; R420Q, 12.5 ± 0.9 Hz). APD_{90} was measured during the first sinus beat following rapid pacing and during steady sinus rhythm.

Optical mapping

Mouse hearts were removed and perfused as described above. Baseline ECGs during sinus rhythm

were recorded before dye loading. For optical V_m recording, hearts were loaded with a 1 mL bolus of di-8-ANEPPS (50 μg/mL) injected into the sideport of the Langendorff perfusion cannula over 5 min. For simultaneous Ca^{2+} and V_m monitoring, hearts were loaded with 25 μg Rhod-2-AM dissolved in 2 mL TYR with Pluronic-F127 (final concentration 0.2%) over 10 min, followed by RH-237 (25 μg/mL in 1 mL) over 5 min. Blebbistatin (10 μmol/L) was included in perfusion solutions during experiments to suppress motion artefacts. Excitation light was provided by four 532 nm LEDs passed through 530±10 nm bandpass filters (FBH530-10, Thorlabs) that were positioned to give even illumination of the left ventricle. Di-8-ANEPPS fluorescence was collected through a 600 nm longpass filter (FELH0600, Thorlabs), or for dual emission recordings fluorescence was split using a Cairn OptoSplit with a 638 nm dichroic (DMLP638R, Thorlabs) and through additional 575 ± 25 bandpass (86-952, Edmund Optics) and 700 nm longpass filters (FELH0700, Thorlabs) to separate Rhod-2 and RH-237 signals, respectively. Image series were recorded in 12-bit at 900 Hz using a high sensitivity sCMOS camera (Kinetix22, Teledyne Photometrics). Image stacks were processed using a 3D Gaussian filter reduce high frequency noise and further down sampled by binning to achieve a pixel dimension of 500 μm. Optical mapping data were analysed using the open-source ElectroMap software.[6] In dual Ca^{2+}/V_m optical mapping experiments the external $[Ca^{2+}]$ and ISO were increased to 1.8 mmol/L and 1 μmol/L, respectively.

Ventricular myocyte isolation

Ventricular myocytes were isolated as described previously,[7] by enzymatic digestion with 1 mg/mL collagenase (Type I, Worthington, NJ) and 0.05 mg/mL protease XIV (Sigma Aldrich). The entire ventricle, including left ventricle, right ventricle and septum was coarsely minced and gently agitated at 35 °C in 0.1 mmol/L Ca^{2+} isolation solution, containing in mmol/L: 137 NaCl, 4 KCl, 10 4-(2-hydroxyethyl)-1-piperazineethanesulfonic acid (HEPES), 10 creatine, 20 taurine, 10 glucose, 1 $MgCl_2$, pH 7.4 with NaOH. Cells were centrifuged at 50x *g*, the supernatant discarded, then cells resuspended in Tyrode's solution. Extracellular $[Ca^{2+}]$ was gradually raised in steps of 0.2, 0.5, 1 mmol/L. Isolated myocyte experiments were performed in 1 mM Ca^{2+} Tyrode's solution at 22 or 35 °C as indicated.

Confocal Ca^{2+} imaging

Ventricular myocytes were loaded with the Ca^{2+}-sensitive indicator Fluo-4-AM (5 μmol/L) for 10 min at room temperature, followed by de-esterification and imaging. Electrically-evoked and spontaneous Ca^{2+} release was recorded using 488 nm argon laser excitation and emission long-pass filtered (>500 nm) using a Zeiss LSM880 or Leica SP8 confocal microscope in line-scanning mode (1 ms/line, 0.1-0.2 μm /pixel) and pinhole set to <2 Airy units at 22 °C. GaAsP photodetectors were used to increase the sensitivity of Ca^{2+} spark detection. Ca^{2+} sparks were detected using an automated Ca^{2+} spark detection algorithm described previously.[7,8] To improve the detectability of Ca^{2+} sparks against the increased background fluorescence and noise during Ca^{2+} transient decay, a high-pass filter was applied to recordings prior to LCS detection. Blebbistatin (10-20 μmol/L) was added to the superfusate to minimise motion artefacts. Ca^{2+} transients were elicited either by field stimulation with a pair of Pt electrodes at 1.3x threshold, or by current injection via patch pipette. Ca^{2+} release synchrony was quantified as the variability in time (standard deviation) across the cell to electrically evoked Ca^{2+} release, defined as the time when fluorescence exceeded 5 standard deviations above background (resting) fluorescence. LCS amplitude restitution data were fit with a single exponential association to calculate the time constant for amplitude recovery. Ca^{2+} sparks were considered as being 'late' if they occurred after the peak and before the time to 90% recovery of the cellular average Ca^{2+} transient for each cell, otherwise they were classed as diastolic and were not included in the calculation of LCS frequency. Detected events classed at LCS are indicated on exemplar line scan recordings by white boxes, and diastolic Ca^{2+} sparks are indicated with an asterisk.

Patch clamp electrophysiology

Recordings were made in whole-cell patch clamp configuration using borosilicate glass patch pipettes (typically 1.6-2.0 MΩ) filled with a mock intracellular solution containing, in mmol/L: 120 aspartic acid, 20 KCl, 10 HEPES, 10 NaCl, 5 glucose, 5 Mg.ATP, 0.05 Fluo-4 pentapotassium salt, with KOH added to produce pH 7.2. This pipette solution gave a +10 mV liquid junction potential measured

experimentally using a 3M KCl agar bridge and recordings were corrected for this potential. APs were elicited by 2 ms current injection pulses at 1.3x threshold and AP morphology was analysed in steady state during pacing at 1 Hz. Superfusate solution was maintained at 35 ± 1 ° C or 22 ± 1 ° C during electrophysiology experiments, as indicated. Membrane potential was recorded using an Axopatch 1D amplifier (Molecular Devices), Power1401-3 digitizer, and Signal data acquisition software (version 6.04, Cambridge Electronic Design) or dPatch amplifier (Sutter) with Sutterpatch acquisition software at 10 kHz. SR Ca^{2+} content was measured under voltage clamp at 35 °C by rapid exchange of extracellular Tyrode's solution with solution containing 10 mM caffeine to empty intracellular Ca^{2+} stores and integrating the current backwards in time.[9] Ca^{2+} loading for SR determination was standardised by 5x 100 ms prepulse steps to 0 mV from a holding potential of -40 mV at 1 Hz, followed by a 1 s pause then rapid application of caffeine controlled by TTL pulse. For AP voltage clamp experiments a typical WT (Control AP) or R420Q AP waveform (CPVT AP) was used as the voltage command. For LCS analysis under AP clamp, cells were paced with a standardised loading protocol consisting of a 500 ms ramp to -50 mV from a holding potential of -70 mV, followed by a 50 ms step to +10 mV that was repeated 5 times at 1 Hz, followed by a test pulse using either a Control or CPVT AP. A pacing-pause protocol was used to quantify the prevalence of EADs in isolated myocytes under current clamp. This protocol involved five stimuli at 1 Hz, followed by a 5 s pause, then a final stimulus. The number of cells developing EADs in response to the final stimulus was compared in WT and R420Q cells in TYR and in ISO (100 nmol/L). This experiment was repeated at 22 and 35 °C with similar results (c.f. Fig. 4D, Fig. S5D).

Immunocytochemistry
Cells were fixed in 2% formaldehyde in phosphate buffered saline (PBS) for 10 min, then resuspended in PBS containing 100 mM glycine. Cells were permeabilized in 1% Triton-X100/PBS for 10 min, then washed three times in PBS, then for 30 min in a blocking buffer (5% normal goat serum (Jackson ImmunoResearch ,UK) and 2% BSA in PBS). Cells were incubated for 1h at room temperature with primary antibodies: rabbit polyclonal anti-RyR2 (19765-1-AP, Proteintech, Manchester, UK) and mouse monoclonal anti-caveolin-3 (sc-55518, Santa Cruz, Heidelberg, Germany) (both used at 1:250 dilutions in blocking buffer). Cells were washed three times in blocking buffer then incubated for 1 h at room temperature with fluorescently labelled secondary antibodies: Alexa 488-conjugated goat anti-rabbit IgG (111-545-003, Jackson ImmunoResearch ,UK) and Alexa 647-conjugated goat anti-mouse IgG (115-605-003, Jackson ImmunoResearch ,UK) both used at 1:400 dilution in blocking buffer. Finally, cells were washed three times in PBS, then mounted on glass coverslips (Prolong Gold, Invitrogen). Cells were imaged on a Zeiss LSM710 confocal microscope, with laser excitation at 488 nm and 633 nm, with emission collected 500-600nm and >650 nm.

Mathematical modelling
A mathematical model of a three-dimensional cardiac myocyte with spatially distributed Ca^{2+} release and rabbit electrophysiology was employed to investigate the role of CPVT-like 'leaky' RyR2 properties on repolarization. The original model was described in detail in Restrepo et al. (2008), with further modifications in Terentyev et al. (2014) and in Zhong et al. (2018), and the reader referred there for detailed descriptions of its implementation.[10–12] Briefly, the model consists of ~20,000 diffusively-coupled Ca^{2+} release units, each consisting of 100 RyR2 and 4 L-type Ca^{2+} channels, distributed in a 3D myocyte structure. CRU are diffusively-coupled via cytosolic, submembrane and proximal dyadic compartments and junctional and network SR compartments. Fixed time steps of 0.025 ms were used for ionic current calculations, with finer 0.005 ms steps for calculation of $[Ca^{2+}]$ fluxes within proximal and submembrane spaces. Simulated Ca^{2+} line scans were generated from the cytosolic Ca^{2+} in central CRU passing through the long axis of the myocyte and were output for display purposes at a temporal resolution of 2.5 ms. The bi-directional interaction between Ca^{2+} and V_m is linked by ionic currents and Ca^{2+}-dependent inactivation of L-type Ca^{2+} channels (LTCC) based on rabbit electrophysiology. In some simulations the electrophysiology was modified to produce more 'mouse-like' action potential, or replaced to simulate a human ventricular action potential based on

the Grandi-Bers model[15] (described below), to evaluate how CPVT RyR2 properties might impact repolarization in these species. The modifications to the model used by Zhong et al. (2018) needed to reproduce the simulation data are described below.

RyR2 hyperactivity in CPVT

We followed a similar approach to induce RyR2 hyperactivity as used by [10,12] (to investigate hyperphosphorylation of RyR2 by CaMKII in long QT type 2 syndrome), by increasing RyR2 Ca^{2+} sensitivity and reducing refractoriness in the simulated presence of isoproterenol. Planar lipid bilayer recordings of single RyR2-R420Q channels show increased transitions to multiple subconductance states, resulting in an overall increase in channel activity by ~2x.[13] RyR2 Ca^{2+} sensitivity in simulations was increased accordingly by reducing the half maximal effective concentration (EC_{50}) of RyR2 open probability from 7.14 µmol/L in control to 3.5 µmol/L in CPVT. Refractoriness of Ca^{2+} release in the model depends on the closed-to-open transition rates of RyR2 in the calsequestrin-2 (CASQ2) unbound state (when SR Ca^{2+} is high, $\overline{k}_{p,U}$) and bound state (when SR Ca^{2+} is reduced, $\overline{k}_{p,B}$). Refractoriness was altered by varying the ratio of $\overline{k}_{p,B}/\overline{k}_{p,U}$ from 0.01 for control RyR2 to 0.2 for CPVT RyR2, while $\overline{k}_{p,U}$ was kept constant at 5 ms^{-1}.[10] This change in ratio should approximately double the rate of LCS amplitude recovery,[10] and this was confirmed by measuring the recovery of SR Ca^{2+} blinks while membrane potential was held at -40 mV (Fig. 8D,E). For comparison with experimentally recorded LCS frequency, the mean simulation LCS rate throughout the entire cell during the Ca^{2+} transient decay was scaled to account for the ~2% of cell volume that is normally surveyed by the confocal scan line during line scan recordings.[14] The same RyR2 properties for Control and CPVT were used for all simulations.

'Mouse-like' model: To produce a mouse-like AP, the existing model rabbit electrophysiology was adjusted by: increasing $G_{to,f}$ to 0.5 nS/pF; setting $G_{to,s}$, G_{kr} and G_{ks} to 0; increasing SERCA Ca^{2+} uptake rate 2x; shifting the reversal potential for I_{K1} by 15 mV. These changes resulted in a shorter, triangular AP with an APD_{90} of 85 ms and resting V_m of -71.3 mV for Control simulations during steady pacing at 5 Hz with $[Na^+]_i$ = 10 mmol/L.

Human model: For the human model: the original I_{kr}, I_{Ks}, $I_{to,f}$, $I_{to,s}$ were replaced with the equivalent human formulations taken directly from Grandi-Bers. I_{NCX} and I_{Ca} were kept the same in mouse, rabbit and human simulations, but I_{Ca} density was reduced in human simulations to produce a peak AP-evoked current magnitude of around -5 pA/pF to be comparable to Grandi-Bers. These changes resulted in a steady state APD_{90} of 235 ms and resting V_m of -86.0 mV in Control simulations during steady pacing at 1 Hz with $[Na^+]_i$ = 7.65 mmol/L.

I_{kr} rapidly activating K^+ current

$$g_{kr} = 0.035 \,.\, \sqrt{\frac{K_o}{5.4}}$$

$$xr_{ss} = \frac{1}{1 + exp\left(-\frac{V_{m+10}}{5}\right)}$$

$$T_{xr} = \frac{550}{1+exp\left(\frac{-22-V_m}{9}\right)} \cdot \frac{6}{1+exp\left(\frac{V_m+11}{9}\right)} + \frac{230}{1+exp\left(\frac{V_m+40}{20}\right)}$$

$$\dot{x}_{kr=} \frac{xr_{ss} - x_{kr}}{T_{xr}}$$

$$r_{kr} = \frac{1}{1 + exp\left(\frac{V_m + 74}{24}\right)}$$

$$I_{kr} = g_{kr} \,.\, x_{kr} \,.\, r_{kr} \,.\, (V_m - E_k)$$

I_{Ks} slowly activating K^+ current

$$F_{junc} = 0.11$$

$$F_{sl} = 1 - F_{junc}$$

$$xs_{ss} = \frac{1}{1 + exp\left(-\frac{V_m + 3.8}{14.25}\right)}$$

$$T_{xs} = \frac{990.1}{1 + exp\left(-\frac{V_m + 2.436}{14.12}\right)}$$

$$\dot{x}_{Ks} = \frac{xs_{ss} - x_{ks}}{T_{xs}}$$

$$I_{Ks_{junc}} = F_{junc} \,.\, g_{Ks_{junc}} \,.\, {x_{ks}}^2 \,.\, \left(V_m - E_{Ks_{junc}}\right)$$

$$I_{Ks_{sl}} = F_{sl} \,.\, g_{Ks_{sl}} \,.\, {x_{Ks}}^2 \,.\, \left(V_m - E_{Ks_{sl}}\right)$$

$$I_{Ks} = I_{Ks_{junc}} + I_{Ks_{sl}}$$

$I_{to,s}$ slow transient outward K^+ current

$$x_{to_{ss}} = \frac{1}{1 + exp\left(-\frac{V_m - 19}{13}\right)}$$

$$y_{to_{ss}} = \frac{1}{1 + exp\left(\frac{V_m + 19.5}{5}\right)}$$

$$T_{xto_s} = \frac{9}{1 + exp\left(\frac{V_m + 3.0}{15}\right)} + 0.5$$

$$T_{yto_s} = \frac{800}{1 + exp\left(\frac{V_m + 60.0}{10}\right)} + 30$$

$$\dot{x}_{to_s} = \frac{x_{to_{ss}} - x_{to_s}}{T_{xto_s}}$$

$$\dot{y}_{to_s} = \frac{y_{to_{ss}} - y_{to_s}}{T_{yto_s}}$$

$$I_{to_s} = G_{to_s} \,.\, x_{to_s} \,.\, y_{to_s} \,.\, (V_m - E_k)$$

$I_{to,f}$ fast transient outward K^+ current

$$T_{xto_f} = 8.5 \,.\, exp\left(-\left(\frac{V_m + 45}{50}\right)^2\right) + 0.5$$

$$T_{yto_f} = 85 \,.\, exp\left(-\frac{(V_m + 40)^2}{220}\right) + 7$$

$$\dot{x}_{to_f} = \frac{x_{to_{ss}} - x_{to_f}}{T_{xto_s}}$$

$$\dot{y}_{to_f} = \frac{y_{to_{ss}} - y_{to_f}}{T_{yto_f}}$$

$$I_{to_f} = G_{to_f} \,.\, x_{to_f} \,.\, y_{to_f} \,.\, (V_m - E_k)$$

$$I_{to} = I_{to_s + I_{to_f}}$$

I_{NaK} Na/K pump current

I_{NaK} from Zhong et al. (2018) was modified to use the $K_{m,Nai}$ (11.0 mmol/L) and $\bar{I}_{Nak}$ (1.8 pA/pF) from Grandi-Bers.

$$I_{NaK} = g_{NaK} \cdot f_{NaK} \cdot \frac{1}{1 + \left(\frac{K_{m,Nai}}{[Na^+]_i}\right)} \cdot \frac{[K^+]_o}{[K^+]_o + K_{m,Ko}}$$

$$f_{NaK} = \frac{1}{1 + 0.1245 exp\left(\frac{-0.1 V_m F}{RT}\right) + 0.0365 \sigma exp\left(\frac{-V_m F}{RT}\right)}$$

$$\sigma = \frac{1}{7}\left(exp\left(\frac{[Na^+]_o}{67.3}\right) - 1\right)$$

$$I_{NaK} = \frac{f_{NaK} \cdot \bar{I}_{NaK}}{1 + \left(\frac{K_{m_{Nai}}}{[Na^+]_i}\right)^4} \cdot \frac{K_o}{K_o + K_{m_{Ko}}}$$

The following currents from Grandi-Bers were included in human simulations but not in rabbit or mouse.

I_{Nabk} background Na^+ current

$$I_{Nabk} = G_{Nabk} \cdot (V_m - E_{Na})$$

I_{kp} plateau K^+ current

$$kp_{kp} = \frac{1}{1 + exp\left(7.488 - \frac{V_m}{5.98}\right)}$$

$$I_{Kp_{junc}} = F_{junc} \,.\, g_{kp} \,.\, kp_{kp} \,.\, (V_m - E_k)$$

$$I_{Kp_{sl}} = F_{sl} \,.\, g_{kp} \,.\, kp_{kp} \,.\, (V_m - E_k)$$

$$I_{kp} = I_{kp_{junc}} + I_{kp_{sl}}$$

I_{ClCa} calcium activated chloride current

$$I_{ClCa_{junc}} = \frac{F_{junc} \,.\, G_{ClCa} \,.\, (V_m - E_{Cl})}{\left(1 + \frac{K_{d_{ClCa}}}{Ca_j}\right)}$$

$$I_{ClCa_{sl}} = \frac{F_{sl} \,.\, G_{ClCa} \,.\, (V_m - E_{Cl})}{\left(1 + \frac{K_{d_{ClCa}}}{Ca_{sl}}\right)}$$

$$I_{ClCa} = I_{ClCa_{junc}} + I_{ClCa_{sl}}$$

I_{Clbk} background chloride current

$$I_{Clbk} = G_{Cl_B} \,.\, (V_m - E_{Cl})$$

Other than these changes in RyR2 properties, ion channel formulation and conductance, the model was identical to that described in detail in the Supplemental Materials in Zhong et al. (2018).[10]

**Parameter**	**Mouse**	**Rabbit**	**Human**
$\overline{k}_{p,U}$ ms^{-1}	5	5	5
$\overline{k}_{p,B}$ (Control) ms^{-1}	0.05	0.05	0.05
$\overline{k}_{p,B}$ (CPVT) ms^{-1}	1	1	1
RyR2 EC_{50} (Control) µmol/L	7.14	7.14	7.14
RyR2 EC_{50} (CPVT) µmol/L	3.50	3.50	3.50
$G_{to,f}$ nS/pF	0.5	0.10	0.1144
$G_{to,s}$ nS/pF	0	0.04	0.0156
G_{Kr} nS/pF	0	0.007836	0.035

G_{ks} nS/pF	0	0.20	0.0035
$G_{Na,bk}$ nS/pF	-	-	0.000597
G_{Kp} nS/pF	-	-	0.002
G_{ClCa} nS/pF	-	-	0.0548125
$G_{Cl,bk}$ nS/pF	-	-	0.009
$K_{m,Nai}$ mmol/L	12.0	12.0	11.0
$\bar{I}_{Nak}$ pA/pF	1.5	1.5	1.8
SERCA V_{up} µmol/L ms^{-1}	1.050	0.525	0.525
LTCC prefactor	1	1	0.5
$[Na^+]_i$	10	10	7.65

Table S1. Electrophysiological parameters in mouse, rabbit and human simulations.

Rabbit simulations were paced to steady state at 1 Hz or, in pace-pause simulations, at 2 Hz followed by a 2 s pause with a single extra stimulus. Human and mouse simulations were paced to steady state at 1 Hz and 5 Hz, respectively. Inhibition of SR Ca^{2+} release during AP repolarization was implemented by instantaneously increasing RyR2 EC_{50} to 200 µmol/L.

Computer simulations were performed using the Hawk supercomputing cluster provided by the Advanced Research Computing at Cardiff (ARCCA) facilities at Cardiff University. Simulations ran on Dell PowerEdge R740 servers, with 2 x Intel Xeon Gold 6148 20-core 2 2.4GHz processors, 384GB memory and 2x NVIDIA Tesla P100 16GB PCIe GPU cards.

Statistics

Statistical analysis was performed using Prism (GraphPad, La Jolla, CA). Data were checked for normality using Shapiro-Wilk test. Data with a normal or log-normal distribution were analysed using parametric statistics, otherwise non-parametric alternatives were used. Sample sizes given in figure legends show the total number of cells (n) and hearts (N) used for each group in that experiment. Individual cells isolated from a heart are not truly independent, but are pseudo replicates,[16] therefore we used hierarchical analyses where possible on normally distributed data (nested unpaired t-tests) to account for possible clustering effects in isolated myocyte experiments. Data are presented as mean ± SEM. Exemplar recordings were chosen to reflect the mean result(s) where possible. $P<0.05$ was considered statistically significant. Exact p values are shown above bars on figures where significant differences occurred, otherwise the absence of bars or “ns” indicates no significant difference.

Supplemental Figures and Figure Legends

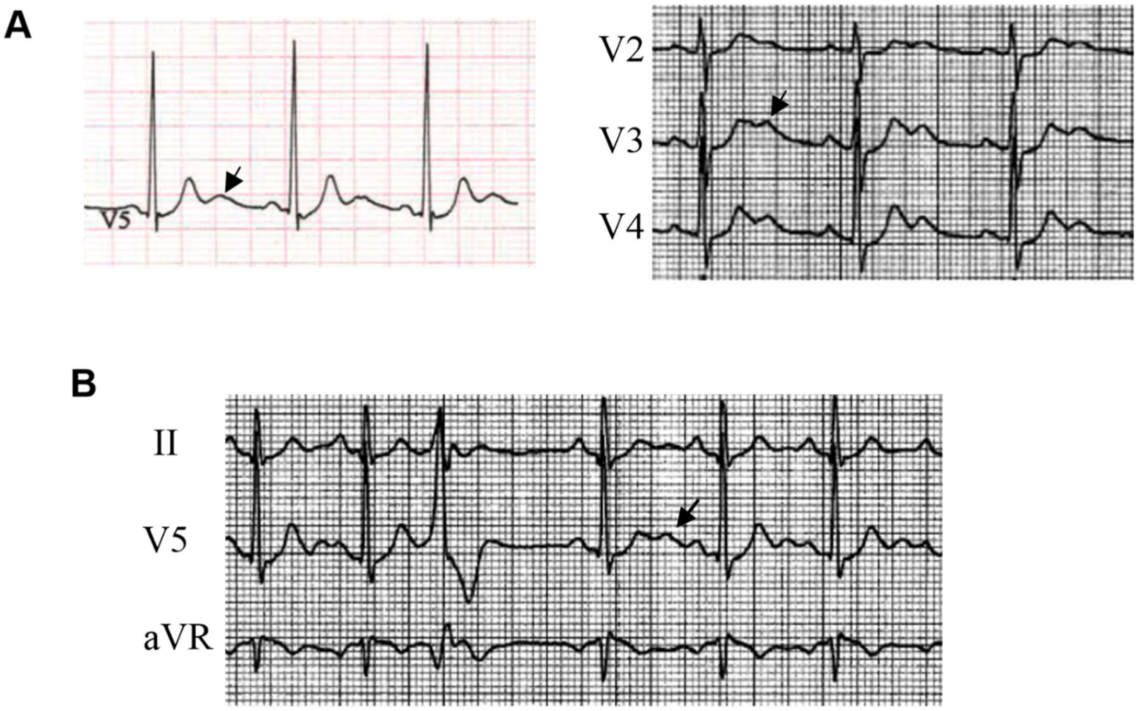

Supplementary Figure 1. A Large U-waves in two human patients with the RyR2-C2277R mutation in the helical domain of RyR2. The U-wave integral in these patients (26.3 and 21.2 mV.ms) was comparable to R420Q patients. **B** Transient increase in U-wave amplitude during the first beat following a post-extrasystolic pause during the recovery period following exercise testing in a patient with RyR2-C2277R mutation. The peak U-wave voltage (black arrow) was comparable to that of the preceding T-wave.

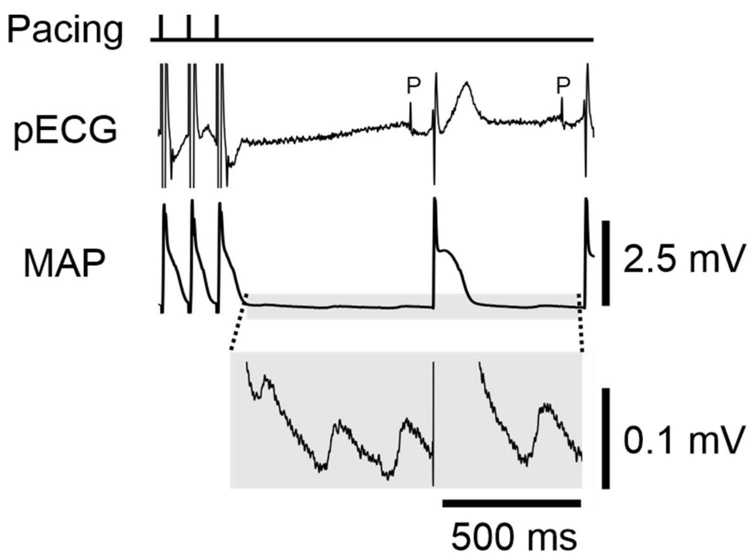

Supplementary Figure 2. Low amplitude depolarizations in the MAP recording during a pause between beats in an R420Q mouse heart might indicate delayed afterdepolarizations (DADs). Despite the presence of DADs, the first beat following the pause was of sinus origin, evident by the presence of a P-wave in the corresponding ECG. DADs were never observed to trigger ectopic beats or VT/VF in any of the isolated R420Q hearts tested under these experimental conditions.

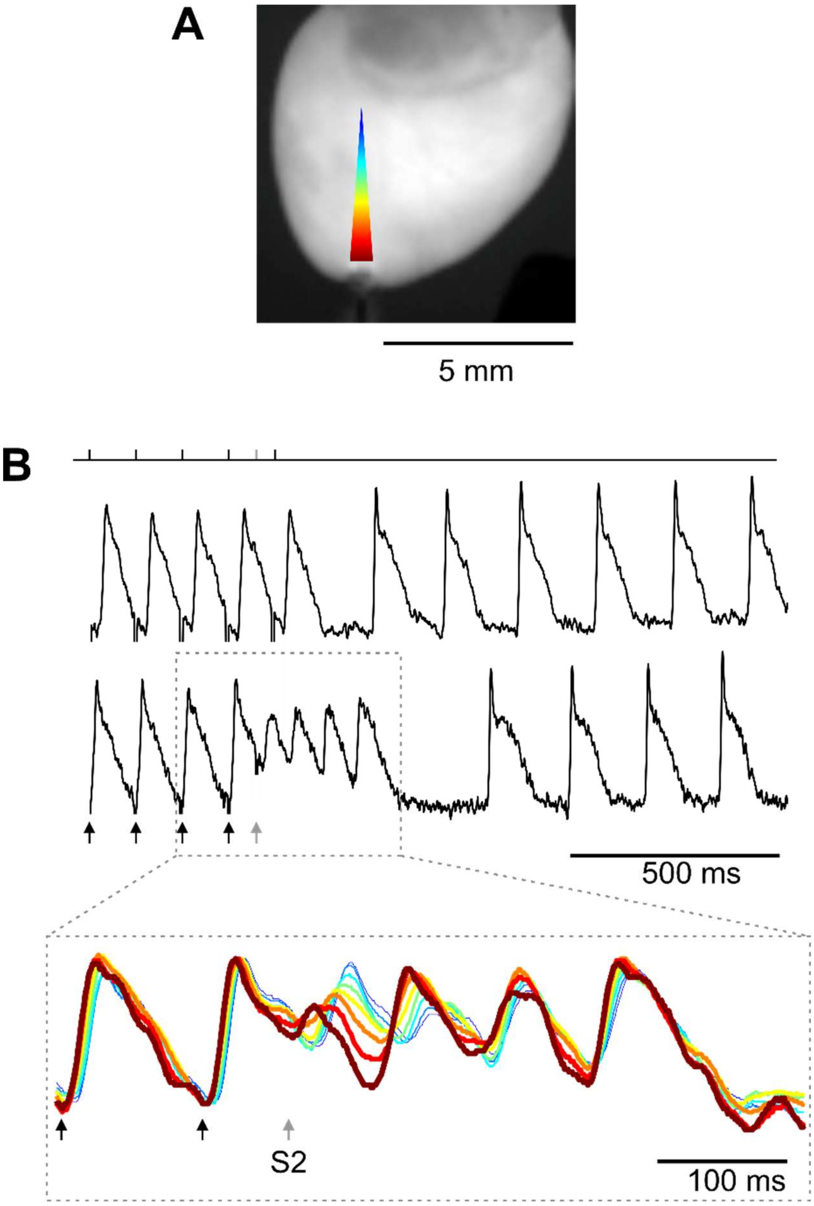

Supplementary Figure 3. Exemplar voltage optical mapping in an R420Q mouse heart during S1S2 premature stimulation protocol in 1 mmol/L Ca^{2+} TYR. **A** Fluorescence image of an R420Q loaded with di-8-ANEPPS. The pacing electrode is visible near the apex. **B** (top) Stimulation protocol consisting of S1 stimuli (at 10 Hz) with the final stimulus at either the normal 100 ms S1 cycle length (black lines), or a shorter 70 ms S2 (grey line). (middle) Mean ventricular fluorescence during S1S1 (middle) and S1S2 (bottom) stimulation. The S2 stimulus induces fast membrane oscillations. Stimulus markers are cropped for clarity. The inset at the very bottom shows the fluorescence of individual pixels along the triangle indicated in panel (A) (from apex to base) during the period of the S1S2 recording indicated by a dashed box in (B). Thicker lines are closer to apex/stimulating electrode, and thinner lines are further away (the line color corresponds to the colors in panel (A)). The initial afterdepolarizations begin out of phase with each other but become more coordinated leading to full repolarization.

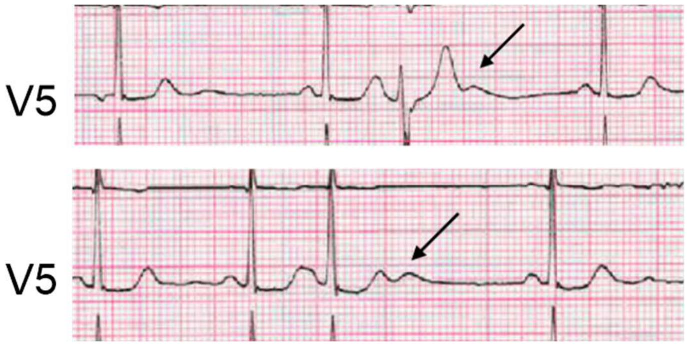

Supplementary Figure 4. Premature ectopic beats in a human R420Q patient were associated with a transient increase in U-wave amplitude.

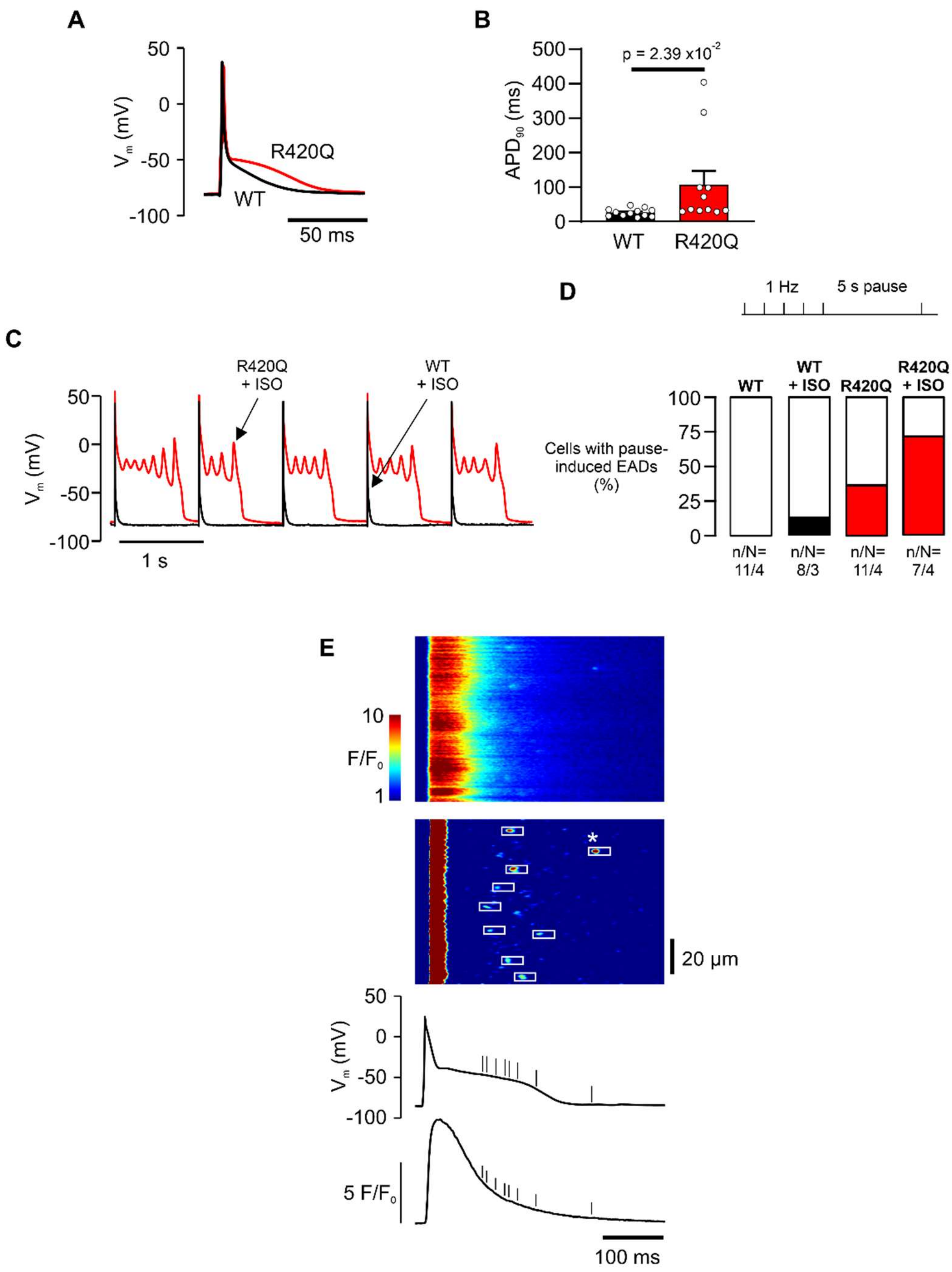

Supplementary Figure 5. Action potential duration prolongation, EADs and LCS occur in R420Q mouse ventricular myocytes at physiological body temperature (35 °C). **A** Exemplar APs during steady pacing at 1 Hz in WT and R420Q cells in TYR at 35 °C. **B** Mean APD_{90} during 1 Hz pacing in TYR at 35 °C. **C** In some R420Q cells treated with ISO (100 nmol/L), spontaneous EADs occurred during 1 Hz pacing at 35 °C. **D** Summary of proportion of cells that exhibited EADs during the final stimulus pulse of a pacing-pause protocol (shown above) at 35 °C. **E** Exemplar Ca^{2+} line scan recording in an R420Q cell during ISO stimulation at 35 °C with clearly visible LCS (event marked * is a diastolic Ca^{2+} spark). Boxes and dashes in lower three panels indicate the location and timing of detected Ca^{2+} sparks in relation to the AP (second from bottom panel) and cellular average Ca^{2+} transient (bottom panel). (B) n/N (cells/hearts) = 12/4 WT and 11/4 R420Q. Nested unpaired t-test. (D) n/N (cells/hearts) as indicated below bars.

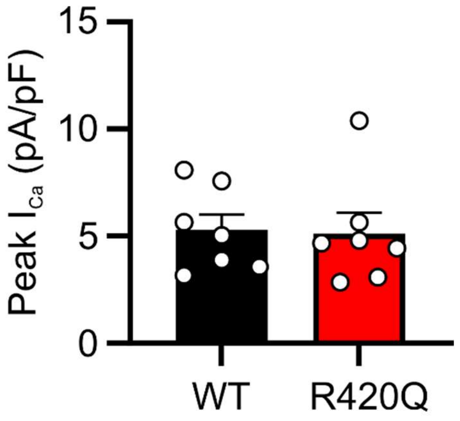

Supplementary Figure 6. Peak I_{Ca} density in WT and R420Q mouse ventricular myocytes. n/N = 7/4 WT and R420Q n/N = 7/3 cells/hearts

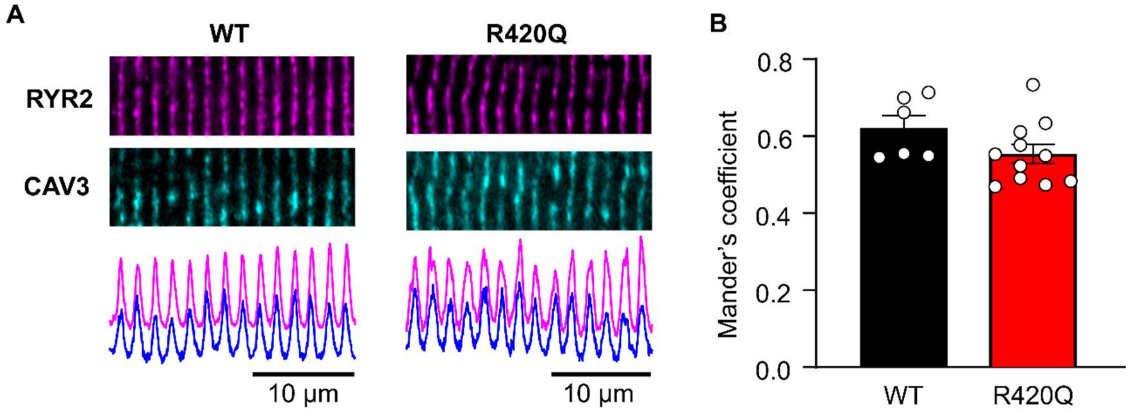

Supplementary Figure 7. Immunocytochemistry showed similar coupling between t-tubules and RyR2. **A** Confocal recordings of a WT (left) and R420Q (right) ventricular myocyte stained with both anti-RyR2 (top panels) and anti-caveolin-3 (CAV-3) (middle panels) antibodies. Lower panels show a high degree of overlap between RyR2 (magenta) and CAV-3 (cyan) signals. **B** Co-localization was quantified using Mander's coefficient and was not different between groups. n/N = 6/2 WT cells/animals and 11/2 R420Q cells/animals. Nested unpaired t-test.

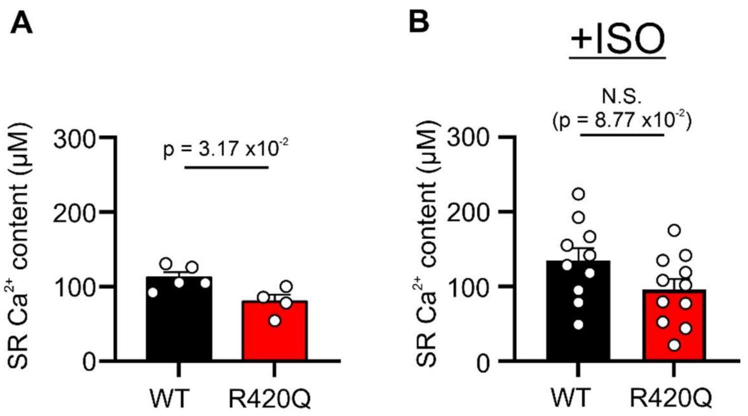

Supplementary Figure 8. Reduced SR Ca^{2+} content in R420Q mouse ventricular myocytes measured by integration of I_{NCX} during rapid application of caffeine (10 mM) in **A** TYR and in **B** 100 nmol/L ISO at 35 °C. (A) n/N = 5/2 WT, 4/2 R420Q cells/hearts. Mann-Whitney t-test, (B) n/N = 10/3 WT, 11/3 R420Q cells/hearts. Unpaired t-test.

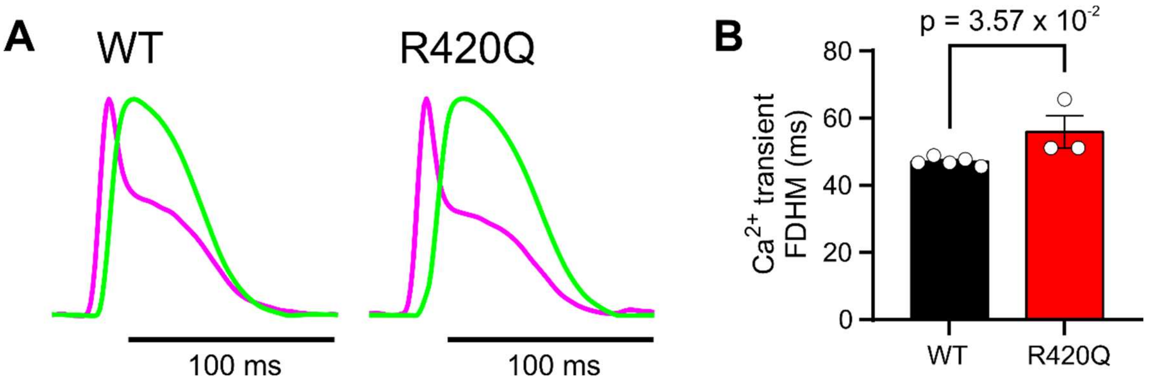

Supplementary Figure 9. Dual Ca^{2+}/ V_m optical mapping in WT and R420Q mouse hearts during sinus rhythm. **A** Exemplar ventricular Ca^{2+} transient (green) and AP (magenta) recorded in a WT and R420Q heart during sinus rhythm in 1.8 mmol/L Ca^{2+} and 1 μmol/L ISO. Shown are the normalized ensemble average APs and Ca^{2+} transients during 8 s recordings. **B** Ca^{2+} transient duration was prolonged in R420Q. N = 5 WT, N = 3 R420Q hearts. Mann-Whitney test

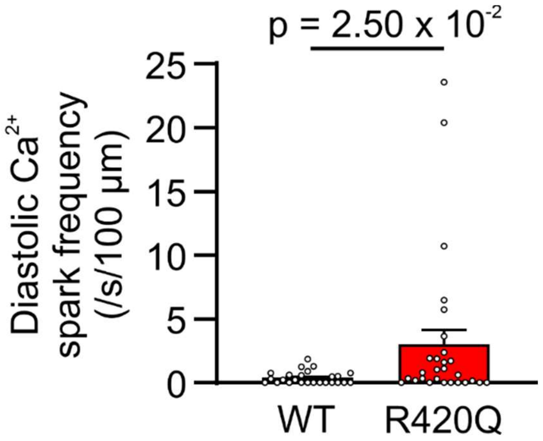

Supplementary Figure 10. Increased diastolic Ca^{2+} spark frequency in R420Q mouse ventricular myocytes in 1 mM Ca^{2+} Tyrode solution. n/N = 25/3 WT cells/animals, 28/3 WT cells/animals. Mann-Whitney test

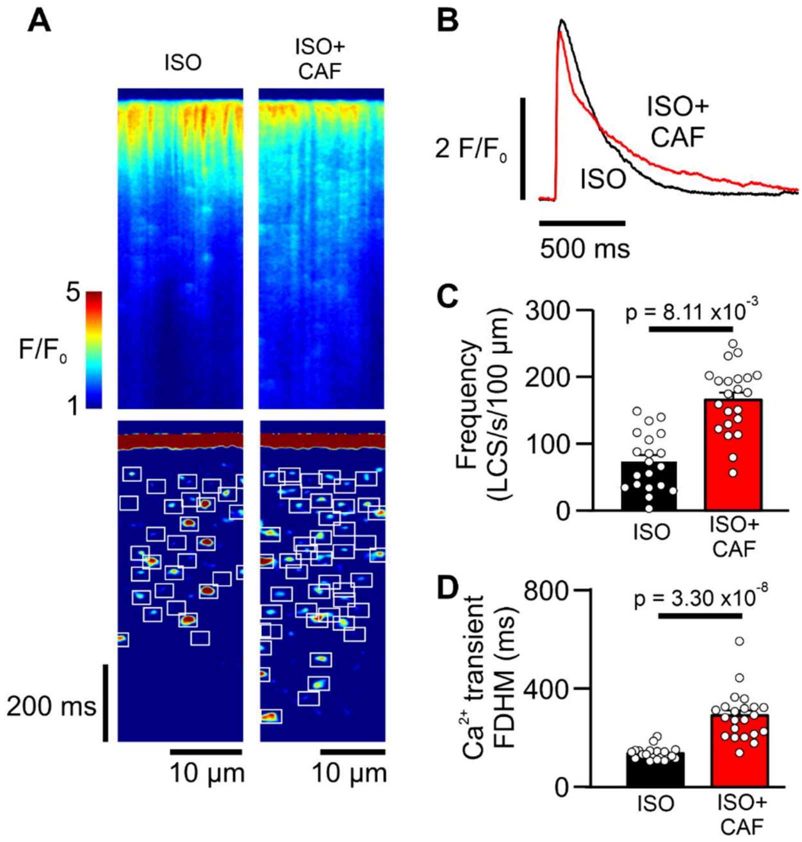

Supplementary Figure 11. RyR2 hypersensitivity promotes LCS and prolongs Ca^{2+} transient duration in WT mouse ventricular myocytes. **A** Exemplar confocal Ca^{2+} line scan recordings of Ca^{2+} transients in WT cells in the presence of 1 µmol/L isoproterenol (ISO) or 1 µmol/L ISO plus 1 mmol/L caffeine (ISO+CAF). All of the highlighted events occur before the time to 90% Ca^{2+} transient recovery and are therefore considered LCS. **B** Whereas the Ca^{2+} transient decayed exponentially in ISO, the decay became biphasic in ISO+CAF and included a prominent slow component, consistent with previous reports.[17] **C** The slow component of decay during ISO+CAF stimulation occurred due to increased LCS frequency that caused **D** increased Ca^{2+} transient duration. (C,D) n/N = 20/3 ISO cells/hearts and 22/3 ISO+CAF cells/hearts. Nested unpaired t-test.

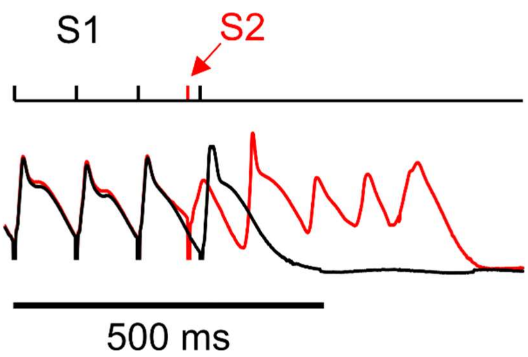

Supplementary Figure 12. MAP recording in a WT mouse heart during perfusion with isoproterenol (100 nmol/L) plus caffeine (100 µmol/L). Steady pacing elicited normal APs (S1, black lines) whereas premature electrical stimulation induced an arrhythmia (S2, red line).

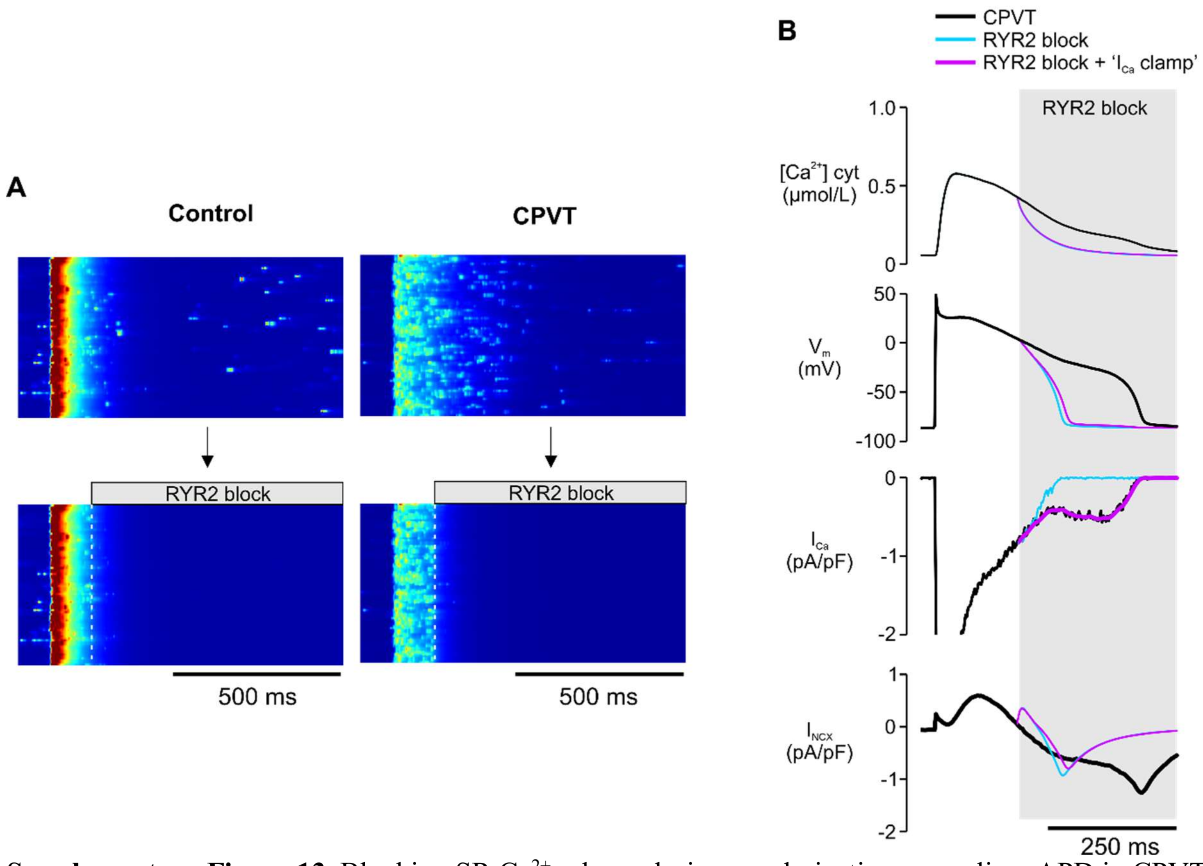

Supplementary Figure 13. Blocking SR Ca^{2+} release during repolarization normalizes APD in CPVT rabbit simulations. **A** Simulated line scans in Control and CPVT simulations during steady pacing at 1 Hz (top panels). SR Ca^{2+} release was blocked by increasing RyR2 EC_{50} to 200 μmol/L at the time indicated by white dashed lines and grey bars, which resulted in an immediate suppression of LCS. **B** (from top to bottom) Cellular average Ca^{2+}, V_m, I_{Ca} and I_{NCX} in the CPVT simulations from (A) without RyR2 block (black lines), with RyR2 block (cyan lines), and with RyR2 block but clamped with the original I_{Ca} profile from steady state CPVT simulations without RyR2 block (magenta). The decrease in SR Ca^{2+} release during the AP reduces inward I_{NCX} magnitude and duration and abbreviates late I_{Ca}, both of which contribute to AP shortening. The small increase in outward I_{NCX} at the onset of RyR2 block occurs due to a fall in subsarcolemmal Ca^{2+} and also facilitates early repolarization. Imposing the original I_{Ca} waveform back in the presence of RyR2 block has a small effect prolonging the AP.

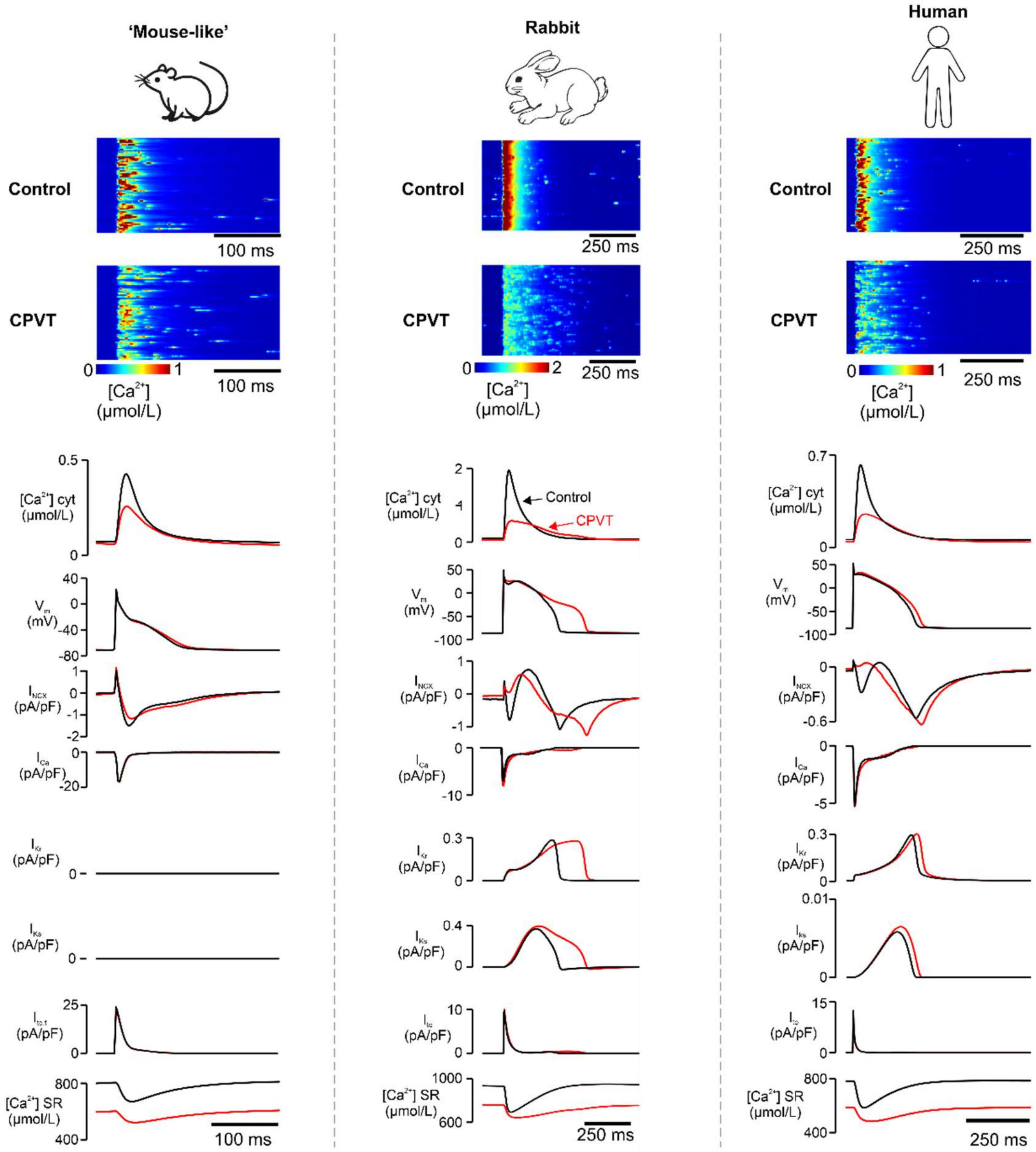

Supplementary Figure 14. Simulations of CPVT RyR2 properties in ventricular myocytes from different species. The structure of ventricular myocytes from mouse, rabbit and humans share many similarities (e.g. t-tubules), but the reliance on membrane ion channels for repolarization differ between species. The 3D cardiac myocyte computer model of spatially distributed Ca^{2+} release was modified to replace the original descriptions of rabbit ventricular myocyte ion channel electrophysiology (middle) with the Grandi-Bers human ventricular myocyte model (right) or modified to produce a 'mouse-like' AP (left) (note the different time scales in mouse vs rabbit & human). Repolarization in mouse is highly dependent on $I_{to,f}$, whereas I_{Kr} and I_{Ks} are dominant in rabbit and human. The Control and CPVT RyR2 properties were the same for all simulations. CPVT increased LCS, slowed the Ca^{2+} transient and reduced its peak, prolonged APD and decreased SR load in all simulations. Reduced Ca^{2+}-dependent inactivation (CDI) of I_{Ca} may contribute to increased late current in rabbit and human but played a smaller role in mouse due to the shorter AP, whereas predominantly inward and prolonged I_{NCX} lengthened APD in mouse. Simulations were paced to steady state at 5 Hz (mouse) or 1 Hz (rabbit, human).

Supplemental References

1. Wang YY, Mesirca P, Marqués-Sulé E, Zahradnikova A, Villejoubert O, D'Ocon P, Ruiz C, Domingo D, Zorio E, Mangoni ME, et al. RyR_2^{R420Q} catecholaminergic polymorphic ventricular tachycardia mutation induces bradycardia by disturbing the coupled clock pacemaker mechanism. *JCI Insight*. 2017;2(8).
2. Klein MG, Krantz MJ, Fatima N, Watters A, Colon-Sanchez D, Geiger RM, Goldstein RE, Solhjoo S, Mehler PS, Flagg TP, Haigney MC. Methadone Blockade of Cardiac Inward Rectifier K+ Current Augments Membrane Instability and Amplifies U Waves on Surface ECGs: A Translational Study. *Journal of the American Heart Association*. 2022;11(11):e023482.
3. Lepeschkin E, Surawicz B. The duration of the Q-U interval and its components in electrocardiograms of normal persons. *American Heart Journal*. 1953;46(1):9–20.
4. Knollmann BC, Katchman AN, Franz MR. Monophasic action potential recordings from intact mouse heart: validation, regional heterogeneity, and relation to refractoriness. *Journal of Cardiovascular Electrophysiology*. 2001;12(11):1286–1294.
5. Curtis MJ, Hancox JC, Farkas A, Wainwright CL, Stables CL, Saint DA, Clements-Jewery H, Lambiase PD, Billman GE, Janse MJ, et al. The Lambeth Conventions (II): Guidelines for the study of animal and human ventricular and supraventricular arrhythmias. *Pharmacology & Therapeutics*. 2013;139(2):213–248.
6. O'Shea C, Holmes AP, Yu TY, Winter J, Wells SP, Correia J, Boukens BJ, De Groot JR, Chu GS, Li X, et al. ElectroMap: High-throughput open-source software for analysis and mapping of cardiac electrophysiology. *Scientific Reports*. 2019;9(1):1389.
7. Fowler ED, Kong CHT, Hancox JC, Cannell MB. Late Ca2+ Sparks and Ripples During the Systolic Ca2+ Transient in Heart Muscle Cells. *Circulation Research*. 2018;122(3):473–478.
8. Kong CHT, Soeller C, Cannell MB. Increasing Sensitivity of Ca2+ Spark Detection in Noisy Images by Application of a Matched-Filter Object Detection Algorithm. *Biophysical Journal*. 2008;95(12):6016–6024.
9. Trafford AW, Díaz ME, Eisner DA. A novel, rapid and reversible method to measure Ca buffering and time-course of total sarcoplasmic reticulum Ca content in cardiac ventricular myocytes. *Pflugers Archiv: European Journal of Physiology*. 1999;437(3):501–503.
10. Zhong M, Rees CM, Terentyev D, Choi B-R, Koren G, Karma A. NCX-Mediated Subcellular Ca2+ Dynamics Underlying Early Afterdepolarizations in LQT2 Cardiomyocytes. *Biophysical Journal*. 2018;115(6):1019–1032.
11. Restrepo JG, Weiss JN, Karma A. Calsequestrin-Mediated Mechanism for Cellular Calcium Transient Alternans. *Biophysical Journal*. 2008;95(8):3767–3789.
12. Terentyev D, Rees CM, Li W, Cooper LL, Jindal HK, Peng X, Lu Y, Terentyeva R, Odening KE, Daley J, et al. Hyperphosphorylation of RyRs Underlies Triggered Activity in Transgenic Rabbit Model of LQT2 Syndrome. *Circulation Research*. 2014;115(11):919–928.
13. Yin L, Zahradnikova A, Rizzetto R, Boncompagni S, Rabesahala de Meritens C, Zhang Y, Joanne P, Marqués-Sulé E, Aguilar-Sánchez Y, Fernández-Tenorio M, et al. Impaired Binding to Junctophilin-2 and Nanostructural Alteration in CPVT Mutation. *Circulation Research*. 2021;129(3):e35–e52.
14. Fowler ED, Wang N, Hezzell M, Chanoit G, Hancox JC, Cannell MB. Arrhythmogenic late Ca2+ sparks in failing heart cells and their control by action potential configuration. *Proceedings of the National Academy of Sciences of the United States of America*. 2020;117(5):2687–2692.
15. Grandi E, Pasqualini FS, Bers DM. A novel computational model of the human ventricular action potential and Ca transient. *Journal of Molecular and Cellular Cardiology*. 2010;48(1):112–121.
16. Sikkel MB, Francis DP, Howard J, Gordon F, Rowlands C, Peters NS, Lyon AR, Harding SE, MacLeod KT. Hierarchical statistical techniques are necessary to draw reliable conclusions from analysis of isolated cardiomyocyte studies. *Cardiovascular Research*. 2017;113(14):1743–1752.
17. Sankaranarayanan R, Li Y, Greensmith DJ, Eisner DA, Venetucci L. Biphasic decay of the Ca transient results from increased sarcoplasmic reticulum Ca leak. *The Journal of Physiology*. 2016;594(3):611–623.
